# A deep learning framework for quantitative analysis of actin microridges

**DOI:** 10.1038/s41540-023-00276-7

**Published:** 2023-06-02

**Authors:** Rajasekaran Bhavna, Mahendra Sonawane

**Affiliations:** 1 Department of Biological Sciences, Tata Institute of Fundamental Research, Colaba, Mumbai, 400005 India; 2 Present Address: Department of Data Science and Engineering, Indian Institute of Science Education and Research, Bhopal, Madhya Pradesh 462066 India

**Keywords:** Dynamical systems, Developmental biology, Software, Biophysics

## Abstract

Microridges are evolutionarily conserved actin-rich protrusions present on the apical surface of squamous epithelial cells. In zebrafish epidermal cells, microridges form self-evolving patterns due to the underlying actomyosin network dynamics. However, their morphological and dynamic characteristics have remained poorly understood owing to a lack of computational methods. We achieved ~95% pixel-level accuracy with a deep learning microridge segmentation strategy enabling quantitative insights into their bio-physical-mechanical characteristics. From the segmented images, we estimated an effective microridge persistence length of ~6.1 μm. We discovered the presence of mechanical fluctuations and found relatively greater stresses stored within patterns of yolk than flank, indicating distinct regulation of their actomyosin networks. Furthermore, spontaneous formations and positional fluctuations of actin clusters within microridges were associated with pattern rearrangements over short length/time-scales. Our framework allows large-scale spatiotemporal analysis of microridges during epithelial development and probing of their responses to chemical and genetic perturbations to unravel the underlying patterning mechanisms.

## Introduction

The apical surface of epithelial cells exhibits specialized actin-rich features. These include microvilli observed on intestinal epithelial cells that are required for absorptive function and stereocilia present in the inner ear for mechanosensing^1,2^. Microridges are another class of actin-based protrusions found on various non-cornified squamous epithelia^3–6^. They form laterally long labyrinthine patterns on the apical domain of peridermal or outer epidermal cell surfaces in zebrafish embryos. Ultrastructural analyses have demonstrated that microridges are comprised of actin filament networks^3,5,7^. Consistently, the function of the Arp2/3 complex is essential for their formation and maintenance^7–9^. Additionally, actin regulators such as cortactin, VASP^8^, Wasl, Cofilin, Eplin, Filamin^7^, and non-muscle myosin-II (NMII)^9,10^ as well as keratin cytoskeleton and Plakin cytolinkers^11^ localize to the microridges. Microridges remain dynamic and actin is actively treadmilling within them^8,10,12,13^. Besides, cell polarity proteins such as aPKC and Lgl—regulators of apical and basolateral domain identity, respectively—control the elongation of microridges^10,14^. These studies provide collective evidence that microridges are organized by F-actin, NMII, and regulated possibly by other actin-binding proteins (ABPs) and cytoskeletal interactions. However, their precise interactions and mechanistic control during formation and maintenance remains elusive. As per the current understanding, F-actin punctae or pegs are distributed on the peridermal cells and apical constriction provides the necessary force for the neighboring pegs to coalesce into microridges^9^, which gradually evolve into labyrinthine patterns, under the influence of Myosin-II activity^7,9,10,13^.

In reconstitution experiments, actin, NMII and their associated motor proteins can be organized into various large-scale patterns^15,16^. The concentrations, kinetic parameters of ATP, and density of actin-related proteins contribute to their collective behavior. Stable, stationary structures can form by self-assembly of actin and related proteins near their thermodynamic equilibrium. In contrast, in an active self-organizing system, different mechanisms can arise, in which the assembled structures reach an active steady state without reaching thermodynamic equilibrium, as energy is continuously consumed and dissipated^15,16^. In vivo microridges remain in a non-equilibrium steady state by continuously reorganizing their patterns constituted by an active network of F-actin, NMII, and related proteins. One mode of understanding the mechanism of microridge formation and maintenance is to gain insight into their physical properties that are both reflective of and contribute towards the process of self-organization. Spatiotemporal fluorescence imaging, segmentation, and tracking form powerful approaches to gain quantitative insight into biophysical properties, including morphological and dynamic characteristics.

Quantitative descriptions of dynamic processes require high-quality image data followed by appropriate analysis methods^17–19^. A major challenge for biologically relevant parameter extraction is image segmentation that correctly identifies pixels within the images. Depending upon image-content and morphological features such as cell membrane, nuclei, or actin filaments, their size, shape, density, and data dimension, rigorous image-based techniques are tailored to accurately address specific tasks and involve many carefully fine-tuned control parameters^20–23^. Often segmentation errors at small spatial scales can yield downstream errors leading to noisy results^24^. One way to circumvent this problem is by training machine-learning models on feature vectors extracted from annotated ground truth data representing all possible variabilities^25–28^. Another subset of machine-learning algorithms are the deep learning methods that utilize the general principles of learning and, in this process, learn data representations with multiple levels of abstraction to discover intricate patterns required for detection or classification. They have remarkably outperformed feature-extraction-based algorithms and surpassed human performance for hard problems^28^. Specifically, convolutional neural networks (CNN) are designed to process multidimensional data arrays, including multi-channel or temporal data sequences^28,29^. They have been applied with success for biological-image analysis for cell type classification^30^, protein subcellular localization^31^, image restoration^32^, and cell segmentation^33–36^.

We designed a fast and accurate large-scale CNN-based quantitative framework for the analysis of microridge patterns. We demonstrate that CNNs are adaptable and scalable to produce trained segmentation models that can serve as high-end feature extractors. From the experimental data, we estimated the bending rigidity and population-level length-scale parameter of microridges. Our flow analysis elucidated the time-dependent accumulation and dissipation of mechanical stresses within the underlying networks of microridge patterns. Our computational analysis of mobile high-intensity actin clusters revealed their influence on localized pattern rearrangements. Importantly, the framework allows a quantitative analysis of microridges, unraveling their mechanism of formation and maintenance at different developmental stages, response to perturbations and diverse genetic backgrounds.

## Results

### Live imaging of zebrafish epidermis and image processing pipeline

**Fig. 1:**
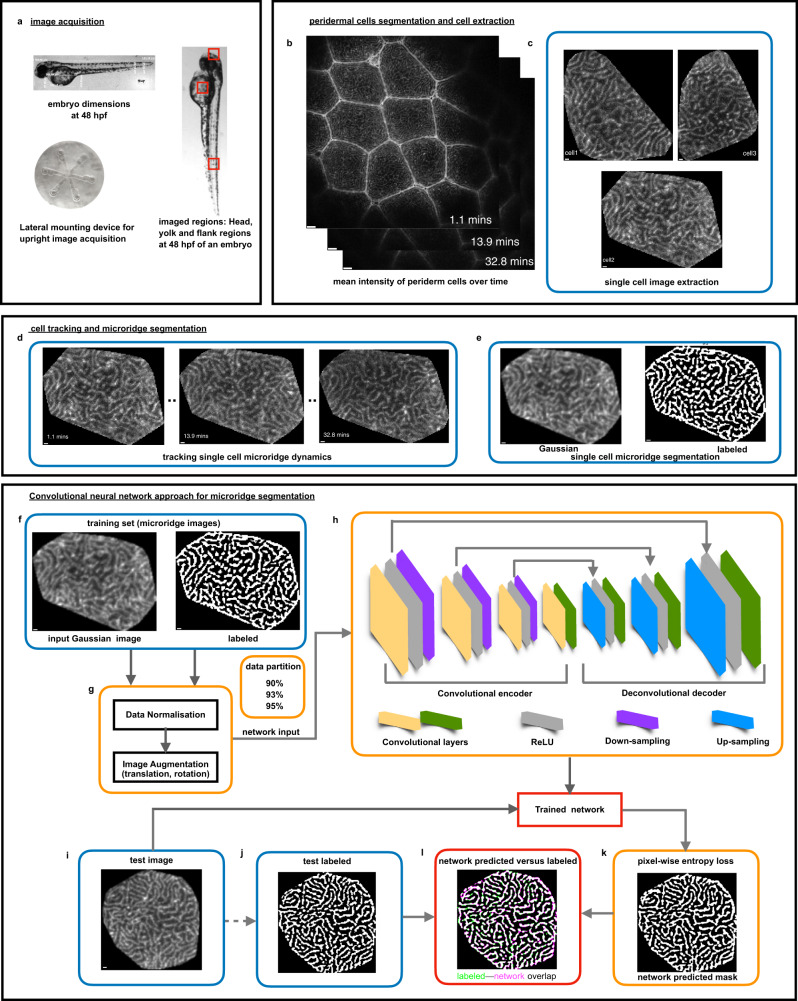
Live imaging, image processing pipeline for a neural network approach for microridge segmentation. **a** Zebrafish embryo dimensions were measured at 48 hpf and a custom-built embryo mounting device was designed for live image acquisition of one lateral side of the head, yolk, and flank embryo regions. **b** Mean intensity of the filtered periderm cell slices at all time points. **c** Membrane segmentation steps lead to demarcated cell boundaries and single-cell extraction. **d** Nearest centroid distance-based cell tracking allowed following each cell’s microridge dynamics. **e** Fully-automated custom-built microridge segmentation algorithm formed the labeled set for the deep learning segmentation strategy (Supplementary Fig. 1, Methods). **f** Convolutional neural network for microridge segmentation. The training set consisted of pairs of extracted cell patterns and their binarized images, illustrated in (**b**, **e**). **g** Prior to training, data normalization and data augmentation steps were implemented. Data were randomly partitioned into 90, 93, and 95% of the total set and various combinations of hyperparameters are trialed in the training process. **h** The convolutional encoder-decoder architecture consisting of a convolutional encoder and decoder layers (yellow and green), ReLU layers (gray), downsampling (purple), and upsampling layers (blue) yielded a trained network for each set of hyperparameters. **i**, **j** The network accuracy was assessed on the remaining test dataset (10, 7, and 5%, respectively) by pixel-wise comparison of network predicted and labeled outputs. **k** Trained network predictions on test data using pixel-wise entropy loss. **l** Labeled versus network-predicted outputs for assessing the network performance. (Scale bars indicate 1 pixel as 0.1977 μm in **b**–**e** and after image re-sizing for CNN to be 0.098 μm in **f**–**k**).

The zebrafish epidermis is bi-layered by 48 hpf with the apical surface of outer peridermal cells decorated with microridges (Supplementary Fig. 1a). To facilitate high-quality live imaging of developing microridges (*Tg(actb1:GFP-utrCH)*), we designed a custom mounting device to fit zebrafish embryo dimensions at 48 hpf (Methods, Fig. 1a). Microscopy parameters were optimized for achieving high spatiotemporal resolution of the epidermis from head, yolk, and flank regions of the embryo. Temporal changes in periderm cell height were sensitive to tissue regions and embryonic development. Therefore, optimal filtering parameters were set for each microscopy movie to encompass peridermal cells and exclude basal epidermal cells from the segmentation analysis (Fig. 1b and Supplementary Fig. 1a–c).

Custom algorithms were written for periderm cell-membrane segmentation to demarcate each cell by its boundary and extract raw single cells patterned with microridges (Methods, Fig. 1c and Supplementary Fig. 1d–g). The cell segmentation steps excluded cells with incomplete edges (membranes) due to either low contrast or non-uniform z-fluctuations. This was followed by cell centroid distance-based tracking frame-by-frame (Fig. 1d and Supplementary Fig. 1h). Cell-tracking information was used for dynamic pattern analysis. We designed an automated microridge segmentation pipeline (Methods, Fig. 1e, Supplementary Fig. 1i) that formed the labeled training set for the CNN approach. The patterned-cell extraction step provided a large training dataset for CNN-based microridge segmentation (Fig. 1f). Mathematical details of cell segmentation, cell tracking, and microridge segmentation are described in Methods (Eqs. 1–7).

### CNN-based microridge segmentation: tuning the training and performance evaluation

A step-wise description of the CNN microridge segmentation workflow is provided below (detailed in Methods). Using the training set, we optimized the hyperparameters (Fig. 1g) to achieve a trained network for microridge segmentation by implementing a U-net encoder-decoder neural network architecture (Fig. 1h) that has already demonstrated its efficiency in the bio-medical segmentation field^33,34^.

#### Training set

The training set consisted of image pairs of grayscale cell patterns and their corresponding annotated images produced with the automated microridge segmentation pipeline. The dataset formed an excellent repository for training the network as the number of pixels that amounted to foreground and background varied across different patterns. These, in turn, served to determine the optimal set of hyperparameters to achieve pixel-wise segmentation (Fig. 1f–h).

#### Optimizing hyperparameters

We applied median pixel image normalization to balance the weight of foreground and background pixels during training to solve the binary pixel-classification problem. Having produced microscopy data within the laboratory, a data augmentation step ensured training diversity to improve the learning process (Methods, Fig. 1g). The network performance was sensitive to hyperparameters. After initial testing of several combinations of hyperparameters, we fixed the image size to 256^2^ (pixels), which scales to the receptive field size and encoder depth of 6 and adjusted the learning rate to 10^−4^. Smaller receptive fields led to higher numbers of falsely classified pixels, whereas optimal performance was obtained at the cost of longer training time. We varied the mini-batch size (MBS) that indicates the subset of data from the training set that is used at each training iteration and maximum epochs (ME), which is the number of iterations through the entire training dataset during the training.

#### Network setup for training

We performed numerical tests by varying (i) the fraction of the training dataset (95, 93, and 90%) and ii) MBS and ME for each data proportion (Fig. 1g, h). The trained network performance was evaluated by measuring the accuracy on the remaining grayscale test images (5, 7, and 10%) whose image pixels were assigned independently by the microridge segmentation pipeline (Fig. 1i, j). The network training time varied from 12–20 h depending upon the combination of hyperparameters (e.g., training time increased with larger ME on a GPU-enabled high-performance cluster.

#### Performance evaluation

**Fig. 2:**
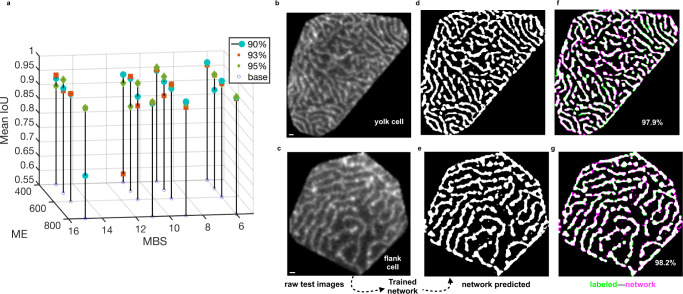
Trained network selection based on predicted accuracy versus network hyperparameters and visual inspection of pixel-wise segmentation. **a** 3D stem plot shows how the mini-batch size (MBS) and maximum epoch (ME) affect the network performance, measured by the mean IOU. For each proportion of the training set, the MBS and ME values were varied as (6, 9, 11, and 15) and (400, 500, 600, and 800) to yield 16 combinations of these hyperparameters. Typically, better accuracy is achievable for smaller MBS and a larger ME, for which our tested combinations of hyperparameters showed performances above ~90% mean IOU. **b**, **c** An exemplary single yolk and flank pattern (1 pixel is 0.098 μm and 0.08 μm), respectively, fed to the selected trained network. **d**, **e** Trained network-segmented outputs for MBS = 6 and ME = 800 using the 93% training dataset (1397 randomly chosen microridge patterns). **f**, **g** Pixel-wise overlap between images labeled using the conventional microridge segmentation pipeline and network-segmented images, shown in green pixels and magenta pixels, respectively; common regions in white (microridges) or black (background). The performance measure is given by mean IOU for each cell pattern.

For each set of hyperparameters, we evaluated the segmentation accuracy by pixel-wise comparison of labeled test images with the network-predicted pixel-wise entropy loss (Fig. 1j, k). The mean intersection over union^37^ (mean IOU) score (Methods) assessed the overall segmentation performance (Fig. 1l). For most cases, an accuracy of about ~90% was achievable. The numerical tests indicated higher segmentation accuracy for smaller MBS and larger ME rather than otherwise (Fig. 2a), suggesting that a repeated learning process was better than giving a greater number of images in one iteration for such a segmentation task. We selected the network trained with 93% data proportion, MBS = 6 and ME = 800 that yielded “mean IOU” of 95.2% (Fig. 2a) on the test data, indicating a reasonable performance (Fig. 2b–g). The microridge segmentation per cell was achieved within a minute on a GPU device using the trained network. The pixel physical sizes (μm) of network-segmented images were re-computed (Methods, Eqs. 8–10) prior to quantitative analyses of their static parameters and their steady state dynamic analysis.

#### CNN versus microridge labeling algorithm

The choice of noise filters and their parameters depends upon the noise type and levels within the acquired images^21^. All images were acquired with a similar signal-to-noise ratio (SNR), and hence the same control parameters for the labeling algorithm (Methods, Eqs. 1, 4) were used. To demonstrate that CNNs can be more robust than the labeling algorithm, we examined the trained network on images acquired at different SNR (Supplementary Fig. 2a, b). The trained CNN segmented these satisfactorily (Supplementary Fig. 2c, d), while the labeling algorithm required fine-tuning parameters to avoid over-segmentation and missing labels (Supplementary Fig. 2e–h).

We then artificially produced high and low-contrast grayscale images, respectively preserving microridge morphology, while altering local pixel intensities (Methods, Supplementary Fig. 3a–c). The de-noising steps were separately modified for the two contrast-type images to produce microridge annotations, since the default approach produced under-segmentation (Supplementary Fig. 3d–g). The previously trained network produced only 30–35% Jaccard similarity on the contrast-altered images (data not shown). One single CNN trained on a contrast-altered dataset containing 3004 cells reported a mean IOU of 84% (Supplementary Fig. 3h–p). Broadly, trained CNN models are relatively more robust than the conventional labeling approaches, particularly when handled by non-experts.

### Estimation of bending rigidity of microridges in vivo

Deciphering the chemo-mechanical properties of microridges is pertinent to understanding the emergence and maintenance of their patterns. We estimated their effective persistence length (*L*_*p*_) or the characteristic length scale at which a microridge bends, to assess their inherent mechanical stiffness. The mechanical responses such as bending, stretching, or compression of the in vivo microridges is likely to be a consequence of active fluctuations in actomyosin network forces, in addition to their dynamic response to thermal fluctuations. Previously, such measurements have been established in in vitro single actin filaments and microtubules, purified extracts of DNA polymers based on their thermal response^38,39^, and microtubules within cells as a consequence of both thermal response and cytoskeleton elements^40^.

**Fig. 3:**
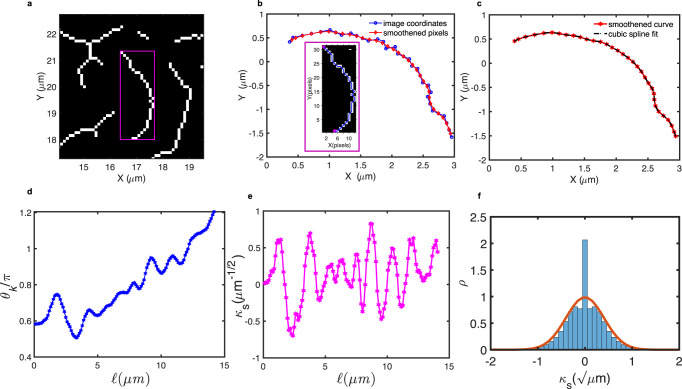
Estimation of persistence length for in vivo microridges. **a** A magenta box demarcates the 2D sub-image of a skeletonized microridge branch for estimation of L_p_. **b** Microridge skeleton contours (blue) were smoothened using a Gaussian fit (red curve). The inset shows a microridge skeleton (blue line) with the endpoints of the contour (magenta) used to obtain the boundary trace that returned the discrete x–y coordinates. **c** A cubic spline interpolation on the Gaussian smoothened microridge trace contours preserved the sequence of points to give several intermediate points. **d** Tangent angle (*θ*_*k*_) along the length (*ℓ*) of the microridge. **e** Rescaled *κ*_*s*_ along the length (*ℓ*) of the microridge contour. **f** Distribution of *κ*_*s*_ of microridges from 1052 cells (293, 1084, and 125 from the flank, yolk, and head, respectively) fitted to a Gaussian distribution (red line trace), whose variance gives an estimate of the effective persistence length (*L*_*p*_) as ~6.1 μm.

We defined an effective *L*_*p*_ based on the overall curvature distribution of microridges (Methods). For each skeletonized microridge branch, (Fig. 3a, highlighted within a magenta box), we obtained discrete x–y pixel coordinates traced along the skeleton contour length (*L*_*xy*_) (Fig. 3b inset) smoothened with a Gaussian, followed by spline interpolation (Methods, Eq. 11–13, Fig. 3c). For each curved segment, we computed the spacing (Δ*s*_*k*_*)* between adjacent points and the tangent orientation angle (*θ*_*k*_) (Fig. 3c), which together allowed the evaluation of the corresponding local curvature (*κ*) (Fig. 3d) along the contour length (Eqs. 14–18). We rescaled the curvature (*κ*) by the square root of the segment lengths to obtain *κ*_*s*_ (Fig. 3e, Eq. 19) and fitted a Gaussian into the distribution $({\mathcal{P}}({k}_{s}))$ (Eq. 20, Fig. 3f), whose width defines an effective *L*_*p*_ estimated as ~6.1 μm. The effective flexural rigidity (Eq. 21) or bending rigidity of microridges was determined as 2.52 × 10^−14^ Nm^2^. The internal forces generated by actomyosin networks within the microridges govern their response under active load, such as stretching, compression or even buckling. For an isolated microridge of length *L* ~1 μm, with the estimated *L*_*p*_, under active load, the critical force (*f*_*c~*_ *π*^*2*^*k*_*B*_*TL*_*p*_*/L*^*2*^) would be about 0.25 pN, above which the microridges would readily buckle. This critical force is less than for pure actin filaments that have *L*_*p*_ ≈ 17 μm^38^ and thus *f*_*c*_ of around 0.69 pN. Our analysis is based on the mesoscopic properties of the network within the microridges. A bottom-up approach, such as building in vitro (or reconstitution) models, would require mimicking the meshwork properties of microridges. Hence, an estimate of *L*_*p*_ describing the mechanical properties of the molecular network is fundamental to probing the role of constituent proteins and deciphering their mechanisms.

### Distinct population level microridge pattern length-scales

**Fig. 4:**
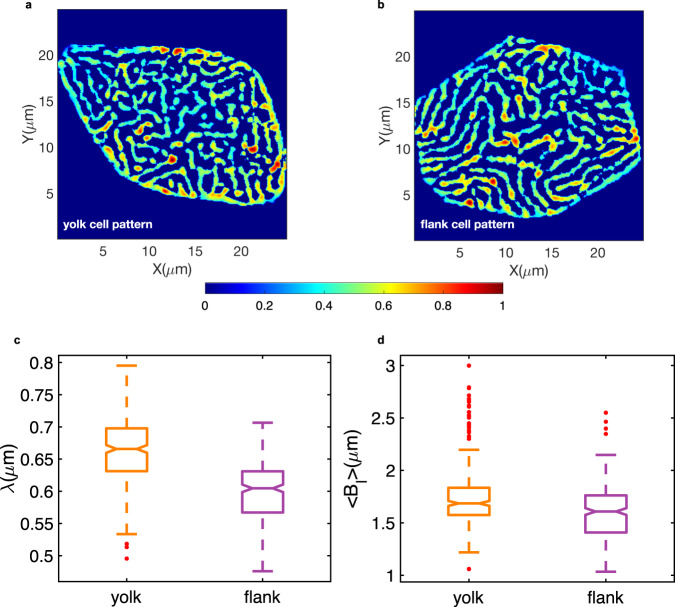
Population level comparison of cell patterns from yolk versus flank regions. Example of a network segmented **a** yolk pattern **b** flank cell pattern, both shown in false color representing image intensities scaled between 0–1, indicated by the colorbar. **c** Box plot of pattern wavelength (*λ*) parameter with estimated medians of 0.66 and 0.60 μm measured from network segmented binary cell images of yolk and flank regions computed over 300 yolk and 293 flank cells, respectively. **d** Box plots of yolk and flank cell population mean microridge branch lengths (<*B*_*l*_>) per cell yielding median values of 1.68 μm and 1.60 μm, respectively quantified from their skeletonized microridge branches.

The emergent pattern of microridges is a signature of their underlying molecular determinants. The concentration, diffusion, and degradation rates of actin and interacting proteins can affect the spacing, density and thereby the pattern length-scale and pattern span characterized by the pattern wavelength. We estimated the wavelength (*λ*) in the Fourier domain (Methods, Eqs. 22–26) for cell patterns from both yolk and flank regions (Fig. 4a, b indicate representative patterns respectively), yielding median values of 0.66 and 0.60 μm, respectively (Fig. 4c). The spatial arrangements of cell patterns from yolk and flank regions were visually discernible with yolk cell patterns being more crowded, comprising of several short-length microridges within the center and longer microridges along the cell periphery, whereas flank cell patterns typically comprised of less crowded but longer microridges. We determined microridge mean branch lengths (<*B*_*l*_>) for each patterned cell (Methods) and found relatively less variance within the yolk population than in patterns from flank regions (Fig. 4d).

### Microridge flow fields revealed the presence of periodic tensile and compressive forces within actomyosin networks

Actin networks driven out of equilibrium by force generation through NMII activity can lead to mechanochemical pattern formation^41–43^. The interplay between active force generation and force dissipation in a viscoelastic environment governs the dynamic properties of such a system and the active stresses could regulate steady flow patterns^41,42^. We performed a spatiotemporal flow analysis of the evolving microridge patterns to gauge the complex bulk dynamics and gain insights into their underlying actin network remodeling process near 48 hpf. We estimated the degree of mechanical stresses due to local deformations by evaluating the pattern velocity flow fields and the corresponding local strain rates. The tracked single cells (Supplementary Movies 1, 2) formed input to the optic flow wherein image intensity flow corresponded to material flow.

**Fig. 5:**
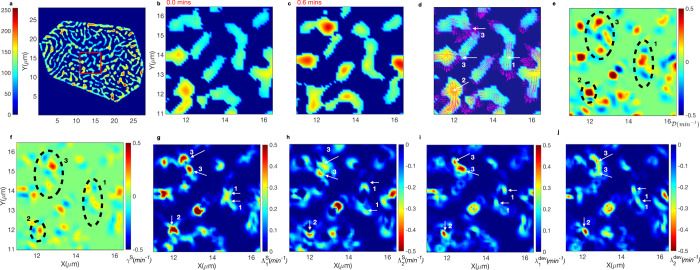
Microridge velocity flow field analysis reveals localized strain rate components within actin-myosin network for generating active flow patterns. **a** A representative network-segmented cell with image intensities shown in false color. A preselected squared center region in red is used to demonstrate the flow analysis. **b**, **c** Microridge pattern at *t*_1_ = 0 min and *t*_1_ = 0.6 min, respectively. **d** Velocity vector field as magenta arrows, indicating the flows from t_1_ to t_2_, shown overlaid on the pattern at *t*_1_. Higher velocities (larger arrows) coincide with either elongation (from within) or fragmentation (at the ends) of a microridge, or merging or splitting of multiple microridges (shrinkage or growth events). White arrows (1–3) indicate preselected regions for a detailed description of divergence and strain rates within the microridges shown in **e**–**g**. **e** Divergence, ${\mathcal{D}}$, shown within the squared region of (a), reaching both large positive and large negative (colorbar), indicating regions with fragmentation or splitting and elongation or merging, respectively. Encircled regions 1–3 indicated regions of elongation, outflows, and inflows, respectively. **f** The shear strain rate (*γ*^*S*^) is shown within the same regions. **g** Tensile strains (${\varLambda }_{1}^{S}$) indicated by arrows 1–3, transiently developed adjacent to regions of elongation or next to outflow or inflow regions. **h** Localized compressive strains (${\varLambda }_{2}^{S}$) adjacent to tensile strains indicated by arrows 1–3, respectively. **i** Deviatoric elongation strains without change in local area (${\lambda }_{1}^{{dev}}$), arrows 1–3, respectively. **j** Strains representing deviatoric shrinkage without change in the local area (${\lambda }_{2}^{{dev}}$), arrows 1–3, respectively.

We selected a square region within a yolk cell center patterned with microridges (Supplementary Movies 1, 3a and Fig. 5a) and analyzed microridge configuration at two consecutive time points, *t*_1_ = 0 and *t*_2_ = 0.6 mins (Fig. 5b, c). The microridge pattern at *t*_1_ superimposed by velocity vectors (magenta arrows) indicates material flows (Eq. 27) between consecutive time points from *t*_1_ to *t*_2_ (Fig. 5d and Supplementary Movie 3b panel-1).

The regions with longer magenta arrows (Fig. 5d) indicated greater movements, signifying active forces arising from molecular interactions driving the dynamic bulk events. These events were: (i) elongations caused by pulling in the outward directions along its length, or steered by fragmentation at the ends of a microridge, either events leading to a change in length of a single microridge, (ii) merging or splitting (similar to fusion or fission^13^) involving several microridges. To examine these events, we computed the velocity field divergence (${\mathcal{D}}$), (Eq. 28, Fig. 5e, and Supplementary Movie 3b, panel-2). High +${\mathcal{D}}$ (red) represented regions with the loss of underlying components within the microridges, hence local flow moving out from a region (fragmentation or splitting events, considered together as shrinkage event), while low -${\mathcal{D}}$ (blue) represented a gain of microridge pixel regions or local flow coming into a region (elongation or merging events, considered together as growth event) between consecutive time points. To focus exclusively on growth or shrinkage events, we determined the 2D-spatial coordinates with large magnitudes of ${\mathcal{D}}$ (|${\mathcal{D}}$| > 0.12 min^−1^) and extracted the velocity magnitudes at such locations. The mean growth velocity (*υ*_growth_) and the mean shrinkage velocity (*v*_shrinkage_) (Methods) were (2.33 ± 1.39) × 10^−2^ μm min^−1^ and (2.34 ± 1.39) × 10^−2^ μm min^−1^ for the yolk and (2.04 ± 1.34) × 10^−2^ μm min^−1^ and (2.05 ± 1.35) × 10^−2^ μm min^−1^ for the flank pattern time series, respectively (Supplementary Movies 1, 2).

The flow analysis provided mesoscopic pointers towards the internal stresses within the actin meshwork, NMII and related proteins involved in complex dynamic flows of actin microridges. In principle, the actin cytoskeleton in the presence of NMII consists of network filaments that could bear both tensile and compressive forces. To examine the local material sources (large +${\mathcal{D}}$) and adjacent sink (large ${\mathscr{-}}{\mathcal{D}}$) regions, we quantified the strain rate tensor (*S*) components (Eqs. 29–31): shear (γ^S^), tensile (${\varLambda }_{1}^{S}$*)* and compressive (${\varLambda }_{2}^{S}$) strains, respectively (Fig. 5f–h). We estimated pure elongation (${\lambda }_{1}^{\mathrm{dev}}$) and pure shrinkage (${\lambda }_{2}^{\mathrm{dev}}$) excluding local area changes (Fig. 5i, j) from the deviatoric tensor *S*^dev^ (Eqs. 32–35).

We compared the same regions within Fig. 5d–j, marked by dashed circles or arrows 1–3. The microridge elongation (arrow-1, Fig. 5d) necessitates two opposite local velocity fields generating two localized ${\mathscr{+}}{\mathcal{D}}$ regions flanked by two adjacently located outer ${\mathscr{-}}{\mathcal{D}}$ regions accompanied by γ^S^ shear strain rates (area encircled by dashed line-1, Fig. 5e, f). The complex internal strain fields exhibited two local tensile strain regions (${\varLambda }_{1}^{S}$) and adjacent compression regions (${\varLambda }_{2}^{S}$), additionally steered by deviatoric elongation (${\lambda }_{1}^{{{\mathrm{dev}} }}$) and deviatoric shrinkage (${\lambda }_{2}^{{{\mathrm{dev}} }}$), respectively (arrows 1, Fig. 5g–j). The localized strains transiently build-up and dissipate immediately in the neighboring region. Next, we examined arrow-2 (Fig. 5d), indicating a merging event of two microridges with outflow velocities in all directions from a single region and hence ${\mathscr{+}}{\mathcal{D}}$ (dashed-circled region-2, Fig. 5e), leading to localized tensile strains (arrow-2, Fig. 5g) and neighborhood compressive strains (arrow-2, Fig. 5h), accompanied by deviatoric shear components (Fig. 5i, j). We observed multiple directed flows within the dashed-circled region-3, arrows-3 (Fig. 5d–j), indicating complex strains within the neighborhood regions. All the flow parameters across consecutive time points for the yolk and flank cell patterns (Supplementary Movies 3b, 4a, b respectively) indicated similar behavior. The microridges exhibited resistive viscoelastic forces alternating between rapid extensions (resistance to outflow) and compressions (resistance to inflow), causing temporal periodic fluctuations. The area-deviatoric decomposition of the S-tensor indicated that deviatoric shear strains, including sliding movements and localized transient area changing events both, contributed to microridge flows.

The overall principal strains (${\left\langle {\left\langle \sqrt{2}{||S||}\right\rangle }_{m}\right\rangle }_{t}$) revealed temporal periodic fluctuations in all cell patterns (Supplementary Fig. 4). We then temporally averaged the principal strains for each cell pattern (Supplementary Table 1) that indicated relatively greater strain fields within the actomyosin networks of microridge patterns of yolk than flank cells (11.15 ± 2.16 × 10^−2^ min^−1^ and 9.77 ± 1.64 × 10^−2^ min^−1^ for Supplementary Movies 1, 2 respectively), suggesting that the stress distributions could be distinctly regulated in different regions of the same embryo.

### Dynamic actin clusters were associated with pattern rearrangements over short length/time-scales

**Fig. 6:**
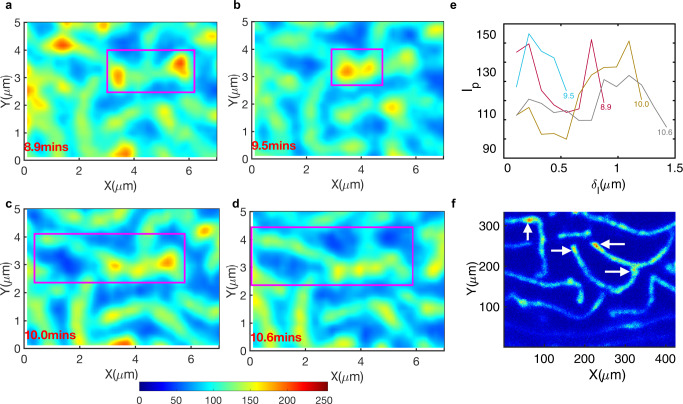
Actin clusters within the microridges exhibit positional fluctuations. **a**–**d** The raw image intensity (in false color) of a representative microridge indicated the presence of actin clusters traversing along the microridge lengths across time frames. **e** Microridge tracking allowed extraction of a one-dimensional intensity profile along the same microridge at each timepoint. Each line color indicates the intensity profile, labeled by the time in minutes, showing the intensity fluctuations within the microridges. High-intensity peaks oscillate in position along microridge lengths. **f** A high-resolution STED imaged with lateral pixel size 0.022 μm in x and y and z-depth of 0.22 μm confirmed the clustered intensity spots within the microridges labeled with Utr-gfp.

Interestingly, we observed high-intensity spots traversing along the microridge lengths, indicating clustered actin speckles exhibiting positional fluctuations. We performed microridge tracking and computationally analyzed the intensity profile along the microridge lengths (Eq. 36). The movement of clustered actin speckles within a microridge (four consecutive time frames, Fig. 6a–d) were quantitatively confirmed by their temporal intensity profile analysis (Fig. 6e) and verified using a high-resolution STED microscope (Fig. 6f), also observed along other microridges (Supplementary Movie 5).

Plausibly, high-intensity clusters become unstable and hence fluctuate in position. This may result in transient and localized spontaneous positive microridge curvatures orthogonal to the cell surfaces. These spontaneous positive curvatures could lead to instabilities and height fluctuations^44^. To analyze the fluctuating intensity spots, we computed the localized Gaussian curvature (*K*), considering microridge intensity profiles to be proportional to their heights (Eq. 37), illustrated within a sub-region of a pattern (Supplementary Movie 6).

We determined the number of 2D-pixel coordinates of high positive and low negative curvatures (|*K*| > 5) and found that the localized high *+K* > *5* constituted ~70% of these, on average, per cell pattern at all time points. We then carried out time evolution of 2D-pixel location coincidence between ±*K* (intensity) and ±${\mathcal{D}}$ locations (significant flows). We found high-frequency counts of *+K* locations at timepoint *t* overlapped with significant flow locations from timepoint *t* to *(t* + *1)* (Supplementary Fig. 5). The positional fluctuations of *+K-*locations in the form of high-intensity clusters continuously altered adjacent sink/source regions within a microridge; consequently, these were associated with localized pattern rearrangements in the form of shrinkage or growth events over short time scales. The short transient time between accumulation and dissipation of mechanical stresses could plausibly influence the formation and mobility of high-intensity clusters at 48 hpf to recruit or retain other ABPs that may influence the turnover and binding constants of subunits of actin. However, the precise molecular cause and the mechanism that drives this process remains to be further determined.

## Discussion

Image segmentation methods are central to transforming live-imaging experiments into quantitative information, which facilitates better understanding of the dynamics of processes of interest. Cell segmentation, tracking, and single-cell extraction from microscopy images are generic methods adaptable for quantitative studies of other cellular processes. CNNs were successfully applied to cell segmentation that requires large labeled datasets typically annotated manually^33,34^. Manual creation of pixel-level annotations for cell surface patterns becomes increasingly laborious and prone to errors. Our automated and flexible microridge masking algorithm alleviates labeler fatigue and increases throughput. We manually verified grayscale cell images with their annotations for segmentation quality prior to the generation of training data. CNNs learn pixel-labels from multiple images by adaptively learning spatial features via self-optimizing their hyperparameters to produce robust segmentation. While altered image quality, either due to acquisition settings or artificially induced contrast, required fine-tuning steps in the labeling algorithm, a single trained CNN model can handle the whole range of image qualities. Once trained, CNNs can serve as high-end feature extractors, making them robust, versatile, and accessible to non-experts. Our CNN strategy consequently improved the data quality required for in-depth quantitative analyses.

The effective persistence length (*L*_*p*_*)* indicated the nature of cytoskeletal networks underlying microridges in vivo, estimated to be ~6.1 μm. Under in vitro conditions, pure microtubules, actin filaments, and DNA show *L*_*p*_ of about 6 mm, 10 μm, and 50 nm and behave as rigid rods, semi-flexible polymers, and very flexible polymers respectively^38,39^. When actin filaments interact with other associated proteins, their *L*_*p*_ is considerably altered. In vitro studies on F-actin and Fascin–actin bundles showed significantly less thermal bending fluctuations in Fascin-associated actin than in F-actin. The rigidity was sensitive to the ratio of F-actin and Fascin bundles, as the stiffness increased with the increasing concentration of actin bundling protein^45,46^. On the other hand, cofilin-decorated actin filaments showed 5-fold lower *L*_*p*_, suggesting greater flexibility than native filaments^47^. Further, actin filaments showed reduced *L*_*p*_ in the presence of phalloidin or heavy meromyosin as compared to bare actin^48^. These studies together indicated that interactions of actin with Myosin or ABPs significantly affected their flexibility and mechanical properties. The overall mechanical properties and the resulting force-related parameters would be modulated by the molecular components^7,8^ within the microridges resulting in lower *L*_*p*_.

F-actin pegs shifted out of equilibrium, possibly due to apical constriction^13^ are driven into a steady state microridge patterning process. Our work suggests additional factors that could influence the evolution of microridge patterns. Our spatiotemporal analysis indicated that 2D planar flows are driven by both tensile and compressive forces and steered by localized shear strain rates present within the microridges at 48 hpf. Temporal periodic fluctuations of mechanical parameters could have implications in mechanochemical feedbacks involving Rho family GTPases^49^ known to drive contractions of the actomyosin cortex in other contexts. Further, we found greater strain fields within the microridge patterns on the periderm cells over the yolk than the flank regions, that can be attributed to various physical and molecular differences present in the two regions. For example, junctional organization on yolk versus flank^50^ does not develop in the same manner, suggesting regional differences in signaling mechanisms. The yolk periderm cells are held at a greater height (from the basal epidermis) and reside over curved surfaces (attributed to overall tissue curvature) in contrast to flank cells. These together, in turn, could rework the underlying active localized stress distributions within the cortex, thereby distinctly remodeling the actomyosin networks and regulate microridge pattern scaling and dynamics. Our comparison of yolk versus flank pattern strain fields revealed that apart from generic features of mechanochemical patterning^9^, the milieu in which cells reside could regulate the stresses within the actomyosin networks.

The low L_p_ indicated that the critical force *f*_*c*_ of microridges is smaller in comparison with other classes of actin-based protrusions. If the protrusive forces of polymerizing actin are not large enough, then such a force could result in microridge growth in the lateral direction rather than causing a significant increase in their heights. Along the lateral directions, we observed and analyzed transient high-intensity actin clusters in the form of spontaneous positive microridge curvatures that exhibited positional fluctuations and traversed at a speed of upto ~2.8 μm min^−1^ along microridges lengths. We anticipated that these unstable clusters influenced the flow events. Concomitantly, we found that 2D-spatial locations of high-intensity clusters coincided with locations of significant flows at consecutive time points, suggesting that dynamic clusters could sporadically lead to either growth or shrinkage events.

Although not determined experimentally, the collective interaction of actin regulators could lead to the formation of spontaneous positive curvatures within microridges exhibiting instabilities and height fluctuations. Recent works have elucidated the role of NMII minifilaments in microridge remodeling by promoting fission and fusion events^13^. Reconstitution studies have depicted a novel picture of NMII minifilaments in organizing various dynamic patterns^15,16,51,52^. NMII activity could drive a network by a multistage coarsening process with NMII foci processively running over actin clusters resulting into a dynamic steady state^52^. This process could lead to actin filaments into disorganized condensates characterized by a broad actin cluster-size distribution^51^. As a result, at certain concentrations, actin clusters subjected to NMII motors may themselves become highly mobile^51^. It is likely that the interaction of NMII motors with F-actin and other actin regulators like Arp2/3 results in dynamic high-intensity clusters within the microridges. The positional fluctuations of actin clusters could be a signature of dynamic re-structuring of the relatively disorganized actomyosin network around 48 hpf, that dictates pattern rearrangements over short length/time-scales. As the pattern matures, microridges organize into a parallel fashion by 96 hpf^13^. Further investigations are required to link the molecular aspects of transient clusters driving actin flows during pattern dynamics.

To summarize, our CNN framework allows large-scale quantitative analyses to decipher the mechanisms of microridge pattern evolution and maintenance. We have identified some of the key aspects influencing microridge pattern dynamics. The persistence length of microridges indicated the range of their force parameters. Our comparative flow analysis elucidated patterns varyingly evolve on different regions of the embryo, suggesting distinct regulation of mechanical stresses within the actomyosin networks. Furthermore, we discovered the transient presence of high-intensity clusters that influenced pattern flows near 48 hpf. Further experimental and theoretical investigations are required to understand the complex interplay of actin, their associated proteins, the role of mechanics, cell morphology, and the role of feedback signaling in shaping the emergent pattern of microridges and their dynamics.

## Methods

### Zebrafish strains

All zebrafish (*Danio rerio*) husbandry and experimental procedures were performed in Tübingen (Tü) strain. We used previously characterized zebrafish lines *Tg(actb1:GFP-utrCH)* provided by Behrndt and colleagues^53^. Zebrafish were raised and kept under standard laboratory conditions. The zebrafish maintenance and experimental protocols used in this study were approved by the institutional animal ethics committee.

### Mounting zebrafish embryos for live image acquisition

Zebrafish embryos were mounted for live imaging to record the dynamics of microridges on the periderm cells from 2–2.5 dpf stages. An agarose-free flat mounting setup, custom fabricated at the TIFR workshop, was used for imaging the lateral side of the embryo. Live imaging was performed in an E3 buffer with 0.02% ethyl-m-aminobenzoate methanesulphonate (Triacane). Live images of the microridges on the periderm cells within the yolk, head, and flank regions of the embryo were obtained using a 40x dipping lens upright Zeiss confocal 880 microscope. The z-stack images from the apical upto basal epidermis were continuously obtained at time intervals of around ~0.5 min for each region during live image acquisition. The images were acquired at a preset room temperature with fluctuations between 25–27 °C. We live imaged eight embryos in 15 different regions (6, 5, and 4 movies on yolk, flank, and head regions, respectively). All images for results in this work were acquired with similar microscopy acquisition settings, with lateral pixel size 0.1977 μm and axial pixel size 0.5–0.6 μm, respectively. To demonstrate CNN robustness, we used additional test images at 1.2 dpf acquired at a lateral pixel size of 0.0988 μm and axial pixel size of 0.5–0.6 μm, thus producing different quality images.

### A custom-built image processing pipeline for microridge segmentation

We describe the details of the image processing pipeline to produce a segmentation mask of the microridges. The algorithm consists of a two-step segmentation approach. In the first, we segmented the periderm cells, followed by cell tracking and frame-by-frame linkage. This was then followed by a tailored segmentation algorithm to label the intracellular pattern of microridges within each cell, enabling a rapid annotation process. The single periderm cell extraction patterned with microridges formed the training datasets for convolutional neural network segmentation.

### Periderm cell image filtering

The images were obtained from periderm cells up to the basal epidermis in the z-direction in XYZT formats (Supplementary Fig. 1a). In order to filter out the periderm slices from the basal epidermis, we implemented a two-step image entropy-based filtering.

The filtering parameter were adjusted separately for cells of yolk, head, and flank images empirically for each confocal image series because the movement along the z-direction (due to tissue thinning along the z-axis) was different within different regions of the embryo and varied across time. Additionally, the yolk cells are relatively taller than the flank cells and this affected how quickly we reached from periderm to basal epidermis in the z-direction. First, the global entropy for each slice was computed using the Shannon information content (Matlab function, entropy), since the slices of periderm cells have relatively lower entropy than the z-slices from the basal epidermis. Therefore, an entropy threshold after a manual inspection was set to filter out periderm slices from the basal epidermis. Secondly, the overlap between the periderm of one cell and the basal epidermis of another cell within the same slice caused signal distortions (primarily in the flank cells). In order to filter out distortions, the local entropy within a 3 × 3 window size (using Matlab function, *entropyfilt*) for the selected slices from the previous step was computed. A pixel-level entropy threshold was then used to filter out noisy pixels due to a signal from the basal that corrupted the microridges and periderm cell membrane. This step was not that crucial for the yolk cell patterns because of their relatively greater cell heights from the basal epidermis. The entropy filtering parameters were set after manual inspection for each confocal image series, and kept constant throughout all time points, to extract periderm cells only (Supplementary Fig. 1b, c). The number of z-planes encompassing just the periderm cells was determined automatically using the entropy information.

### Periderm cell segmentation

The mean intensity along the z-direction of filtered periderm slices was computed and the data for image segmentation was reduced to XYT dimensions (Supplementary Fig. 1d). We used a low pass, linear, Gaussian smoothing filter given by,

1 $$g\left(x,y{\rm{;}}\sigma \right)=\left(1/2\pi {\sigma }^{2}\right)\exp \left[-\left({x}^{2}+{y}^{2}\right)/2{\sigma }^{2}\right],$$

with a standard deviation of σ = 0.7 pixels to reduce noise in all the images.

Next, to demarcate the cell membrane boundary only, the microridges were eliminated (momentarily) from within the cells. For this, we applied a Butterworth high pass (BHP) frequency filter of order *n* and cut-off frequency D_0_ defined as

2 $${\rm{H}}\left(x,y\right)=\frac{1}{1+{{[{\rm{D}}}_{0}/{\rm{D}}\left(x,y\right))]}^{2{\rm{n}}}},$$

where *D(x,y)* is given by,

3 $$D\left(x,y\right)=\sqrt{{\left(x-\frac{r}{2}\right)}^{2}+{\left(y-\frac{r}{2}\right)}^{2}}.$$

Here, *r, c* are the image width and height in pixels, respectively (Supplementary Fig. 1e) with parameters *n* = 1 and D_0_ = 3 (https://www.mathworks.com/matlabcentral/fileexchange/40579-frequency-domain-filtering-for-grayscale-images). This step was followed by image binarization and morphological operations to close any gaps on the cell membrane due to varying image contrasts. The parameters for morphological operators, *imclose* and *strel* Matlab functions were adjusted depending on the image contrast, and then kept constant throughout all time points for a single movie. Each enclosed region (cell) bounded by the cell membrane was masked using the complementary image (*imcomplement* function) as shown in (Supplementary Fig. 1f).

### Single periderm cell extraction

The Area and Solidity properties for each cell enclosed by completed boundaries were computed. We implemented suitable cut-off values on the “cell area” and “solidity” to eliminate cells only partially visible at imaging borders. The cells for which the cell boundaries were incomplete were automatically discarded in this process. For the remaining selected cells, enclosed by completed boundaries, the “BoundingBox”, “ConvexImage”, and “Centroid” properties were computed. The *BoundingBox* finds rectangular coordinates for each enclosed cell region. Each “BoundingBox” coordinate corresponded to a single cell and was used to extract rectangular regions of a cell using the *imcrop* function on the original Gaussian smoothened image consisting of both microridges and cell membranes. This allowed the isolation of each cell with their microridges from the initial images (Supplementary Fig. 1g). Cell shapes are typically polygonal, however, the bounding box coordinates resulted in rectangular selection around each cell. In order to mask the cell enclosed by their membrane, the *ConvexImage* property was computed. The *ConvexImage* is a binary image with only enclosed pixels of a cell bounded by its membrane set to 1’s (Supplementary Fig. 1g). The Hadamard matrix product of the cropped rectangular cell region and its *ConvexImage* resulted in selective extraction of individual periderm cells patterned with microridges over time (Supplementary Fig. 1g).

### Periderm cell tracking

In order to track the same periderm cell frame to frame, a nearest neighbor approach based on the Euclidean distance (cut-off ≤2.5 μm) between cell centroids at time *t*_*n*_ and *t*_*n+1*_ was implemented. The cell tracking allowed us to follow the same cell patterned with their respective microridges as obtained from the microscope (Supplementary Fig. 1h). The images of tracked single cells patterned with their microridge formed the input to the optic flow analysis and the dynamic spot intensity analysis of microridges.

### Microridge segmentation (labeling) algorithm: labeled images for the training set

For each extracted periderm cell, we used a Gaussian smoothing filter (σ = 0.7 pixels) given by Eq. 1 to smoothen the microridges pixels and filter out low background noise from raw cell patterned images. We convolved the resultant Gaussian smoothened image, *IM*(*x*, *y*) with the derivative of Gaussian ∂*g*(*x*, *y*; *σ*_*g*_) with σ_g_ = 0.7 pixels to obtain the microridge intensity gradient (https://www.mathworks.com/matlabcentral/fileexchange/8060-gradient-using-first-order-derivative-of-gaussian),

4 $$\nabla {IM}\left(x,y\right)=\left[{\partial }_{x}g* {IM},{\partial }_{y}g* {IM}\right],$$

where * denotes convolution (Supplementary Fig. 1g).

We computed the second derivative of the Gauss gradient image to obtain the Hessian matrix at each pixel. The trace of the matrix gives the image Laplacian given by,

5 $${L}_{a}\left(x,y\right)=\frac{{\partial }^{2}{IM}}{\partial {x}^{2}}+\frac{{\partial }^{2}{IM}}{\partial {y}^{2}}.$$

We considered only negative Laplacian values *L(x,y)*,

6 $$L\left(x,y\right)=\left\{\begin{array}{c}{L}_{a}\left(x,y\right)\\ 0\end{array}\right.\begin{array}{c}{L}_{a}\left(x,y\right)\le 0\\ {L}_{a}\left(x,y\right)\, > \,0\end{array},$$

in order to select regions with negative intensity emphasizing only on microridges.

Subsequently, in order to obtain a smoothened image segmentation mask, we fitted the *L(x, y)* matrix into a logistic sigmoid function given by,

7 $$S=\frac{1}{1+{{\exp }}\left(-\left|\sqrt{\left|L\left(x,y\right)\right|}\right|\right)}.$$

As a next step, we generated a binary image using the *imbinarize* Matlab function on image *S*. This resulted in a labeled binary image (*B*) of microridges (Supplementary Fig. 1i). The image processing pipeline described so far was used to create a labeled training dataset for neural network pixel-wise semantic segmentation. Pairs of raw extracted cells patterned with microridges and their corresponding labeled image (*B*) formed the training set for the CNN-based microridge segmentation framework.

### Training datasets for convolutional neural network microridge segmentation

The extracted raw microridge cell images formed the input for training the segmentation network. Corresponding annotated images of microridges consisting of foreground pixels (microridges) and the background pixels formed the labeled training set to determine the network parameters. We implemented a semantic segmentation approach, which converts the image segmentation problem into a pixel-level image classification problem. The task was reduced to finding a binary classifier for each image of the training set. Using this approach, we constructed a labeled training set with images of microridges within cells from regions of the yolk, head, and flank of the embryo. We visually inspected each raw cell image with their microridges and eliminated cells with occlusions or very bad contrast on the microridges owed to microscope imaging to maintain high-quality training dataset. Complete manual segmentation was not considered for microridges, owing to their small size and manual errors. We manually checked for segmentation quality by inspecting each pair of grayscale cell image with their annotated labels produced by the microridge segmentation algorithm and discarded any incorrectly segmented cells (~38%) from the analyses. This was much faster than investing in complete manual segmentation to obtain the ground truth, yet it provides a significant manual component to training data generation. For the CNN segmentation, the dataset consisting of raw and labeled image pairs was provided during training (without any cell tracking information).

Several pattern configurations with varying but visually discernible local contrasts were considered for the training set. This enabled the network to achieve better learning on the tested hyperparameters required to discern the pattern with a variety of complexities. The dataset consisted of 1502 single periderm cell images (293, 1084, 125 from flank, yolk, and head, respectively) patterned with microridges. We varied the training set images randomly by partitioning the dataset into 95, 93, and 90% as training set and the remaining as test sets (75, 105, and 150 images, respectively) for all combinations of tested hyperparameters. For our segmentation problem, we used the encoder-decoder segmentation architecture that performed reasonably well on the dataset.

### Hyperparameter tuning for training

We varied the size of the image, which scales to the receptive field size initially. The original 2D microscope image frames were acquired at 512 × 512 pixels for all the cells. For the CNN approach, we trained the network with cell image dimensions of 128^2^, 200^2^, and 256^2^. Similarly, we tested the encoder-decoder subnetwork at depths of 2, 4, and 6. We tested the initial learning rate (*ilr*) for 10^−3^ and 10^−4^. Training of all network models was performed using stochastic gradient descent with *ilr* = 10^−4^, receptive field size set to 256^2^, and encoder-decoder subnetwork depth at 6. We implemented the median pixel-weighted image normalization. A data augmentation approach was included by image rotations, translations, and reflections for all training sets and different permutations of hyperparameters. Obtaining the best-performance network required empirical analysis and hyperparameter tuning. For each proportion of the training dataset (90, 93, and 95%), we varied the mini-batch size (MBS) with values of (6, 9, 11, and 15) and the number of iterations or maximum epochs (ME) with values of (400, 500, 600, and 800) that yielded 16 combinations of these hyperparameters. All the training models were run by creating executables using MATLAB compilers to accelerate the training process. The codes were run on a single GPU (NVIDIA Tesla V100 16GB) node on a high-performance cluster. We confirmed the performance of the trained network with reported accuracy using a test dataset.

### Segmentation performance evaluation metrics

We implemented the standard evaluation metrics for the assessment of image segmentation network performance on test datasets. These included mean pixel accuracy for each class for the entire test dataset, image-wise and class-wise accuracies, and Jaccard similarity coefficient^37^. The mean intersection over union (mean IOU), or Jaccard similarity coefficient was used to benchmark the segmentation performance when comparing results from different combinations of hyperparameters for all proportions of the training set. The Jaccard similarity measures the average IoU score of all classes in all images, and is the ratio of correctly classified pixels by the network to the total number of labeled and predicted pixels in that class. We then evaluated the network performance for MBS and ME for each data proportion.

### Generation of network-segmented cells patterned with microridges

After successful training, the networks generated segmented periderm cells patterned with microridges in the form of binarized images (*NB*). We created a masked cell image (*NM*) given by the Hadamard matrix product of cell patterns (*IM*) and the corresponding network-produced image binary (*NB*),

8 $${NM}={IM}.* {NB}.$$

The masked image (*NM)* is defined on only those gray pixels in the original image where the *NB* image takes values of 1.

### Adjustment of pixel sizes of network-segmented images

All the input images were scaled to dimensions 256 × 256 in xy in order to obtain high performance on the network training. The original microscopy movies were acquired at spatial dimensions 512 × 512, and consequently, original cell sizes were of smaller dimensions. The output of network-segmented microridge cell images (*NM, NB*) were also of size 256^2^ pixels (~2.5 times than the original cell in each dimension). Hence, prior to computing quantitative parameters of microridges, we calculated the physical pixel size in the x and y directions from the original cell dimensions. All confocal images were acquired at pixel size, *psz* = 0.1977 μm in both xy directions. Hence, for an extracted cell image dimension (*x*_*o*_ × *y*_*o*_), we rescaled the pixel sizes ∆*x* and ∆*y* in microns in x and y according to new image sizes of 256 × 256, given by,

9 $${\Delta} x=(x_{o}\,{\cdot}\, {\rm{psz}})/256.$$

10 $$\Delta y=(y_{o}\cdot {\rm{psz}})/256.$$

### Curvature analysis for estimation of microridge persistence length

We describe a method to estimate the distribution of microridge curvature in their dynamical steady state from the skeletonized images of their branches. First, we discuss how to obtain the microridge contour from the images to implement the curvature estimation method. Then we use the experimentally determined curvature distribution to estimate the persistence length and the flexural rigidity that describes the inherent mechanical property (characteristic length scale and bending rigidity) of the microridges.

### Microridge contour and their orientation

We identified the endpoint coordinate pixels for each microridge skeleton. We then traced the skeleton boundary from one endpoint to another to obtain discrete x–y coordinates (*L*_*xy*_) along the length of a microridge skeleton contour. To effectively reduce measurement errors in the estimation of curvature, we implemented a lower frequency curve using Gaussian smoothing fit on the discrete x–y coordinates with a moving window frame = 5 (coordinates) and analysed all the contours with at the least 10 x–y coordinate points to avoid any curve fitting errors. To ensure that the smoothened curves closely followed the skeleton contours for any arbitrary shaped microridge contour, independent of their orientation within the image, we introduced a 2D rotation matrix *R*(*θ*_*R*_) for a given angle, *θ*_*R*_,

11 $$R\left({\theta }_{R}\right)=\left[\begin{array}{cc}{{\cos }}{\theta }_{R} & -{{\sin }}{\theta }_{R}\\ {{\sin }}{\theta }_{R} & {{\cos }}{\theta }_{R}\end{array}\right],$$

where −π ≤ *θ*_*R*_ ≤ π, and rotated each skeleton coordinate *L*_*xy*_ coordinate in the xy plane by an angle *θ*_*R*_ according to

12 $${R}_{{xy}}\left({\theta }_{R}\right)={L}_{{xy}}\cdot R\left({\theta }_{R}\right).$$

The orientation of the rotated skeleton trace ${R}_{{xy}}\left({\theta }_{R}\right)$ now depends on the angle *θ*_*R*_. We varied *θ*_*R*_ over 21 different values and for each, implemented the Gaussian smoothing to obtain a new set of coordinates given by ${L}_{{x}_{k}{y}_{k}}\left({\theta }_{R}\right)$. From these, we selected a single ${R}_{\widetilde{x}\widetilde{y}}\left({\theta }_{R}\right)$ for which the smoothened curve was closest to the skeleton contour, thus realizing,

13 $$\min {{\langle }}D\left({L}_{{x}_{k}{y}_{k}}\left({\theta }_{R}\right),{L}_{{xy}}\right){{\rangle }},$$

where “*D*” denotes the standardized Euclidean distances between all points in ${L}_{{x}_{k}{y}_{k}}\left({\theta }_{R}\right)$ and *L*_*xy*_. We then implemented a cubic spline interpolation method (cscvn function) that preserved the sequence of points to give several intermediate points on the Gaussian smoothened microridge trace contours, thereby reducing the spacing between the data points on the microridge trace contour for estimation of microridge persistence length *(L*_*p*_*)*.

### Estimation of persistence length and flexural rigidity from curvature distribution

The classical approaches for the estimation of *L*_*p*_ include the Fourier shape-fitting method that depends on the number of estimated modes described for actin filaments and microtubules^38^ and the curvature distribution method described for DNA chains^39^. For the microridge data, we followed the curvature distribution method, since the Fourier shape-fitting method requires the right number of modes to be fitted, which may not work for all microridge configurations. For a discrete set of two-dimensional coordinates (*x*_*k*_*,y*_*k*_) along the length of the microridges with (*N* + *1*) points, along the length of the curve (*L*), the spacing Δs_*k*_ between coordinates is given by

14 $${\Delta}{s}_{k}=\sqrt{{(x_{k+1}-{x}_{k})}^{2}+{(y_{k+1}-{y}_{k})}^{2}}.$$

The tangent angle for a set of *N* segments that connect the points is given by,

15 $${\theta }_{k}={{{\tan }}}^{-1}\left(\frac{{y}_{k+1}-{y}_{k}}{{x}_{k+1}-{x}_{k}}\right).$$

The arc length *L* along the microridge length is given by,

16 $$L=\mathop{\sum }\limits_{k=1}^{N}{\Delta s}_{k}.$$

We then estimated *φ*_k_, the angle between two consecutive tangent vectors as

17 $${\varphi }_{k}={\theta }_{k}-{\theta }_{k+1}.$$

The curvature (*κ*) was approximated for small angle changes and small bond lengths at each coordinate over the average arc length of the two adjacent segments by

18 $$\kappa \approx \frac{2\,{\varphi }_{\kappa }}{{\triangle s}_{\kappa -1}\,+\,{\triangle s}_{\kappa }}.$$

In a subsequent step, it will be inconvenient that the segment lengths *∆s*_*κ*_, vary across our ensemble; hence we defined a rescaled variable *κ*_*s*_, such that,

19 $${\kappa }_{s}\approx \frac{2\,{\varphi }_{\kappa }}{\sqrt{{\triangle s}_{\kappa -1}\,+\,{\triangle s}_{\kappa }}}.$$

We then defined the effective persistence length (*L*_*p*_) based on the width of the distribution $({{P}}\left({\kappa }_{s}\right))$ in two dimensions, since the probability of having a certain bending angle follows a normal distribution in thermal equilibrium^39^. The distribution is then given by

20 $${{P}}\left({\kappa }_{s}\right)=\sqrt{\frac{{L}_{p}}{4\pi }}\,\exp \left(-\,\frac{{L}_{p}\,{{\kappa }_{s}}^{2}}{4}\right).$$

The variance of the distribution is inversely proportional to the persistence length (*L*_*p*_). The flexural rigidity $({\rm{E}}{{\rm{I}}}_{a})$, which characterizes the propensity for thermal bending of a flexible polymer^38^ can then be estimated from

21 $${L}_{p}=\frac{{\rm E}{{\rm I}}_{a}}{{k}_{B}T},$$

where *k*_*B*_ is the Boltzmann constant, *T* is the absolute temperature in Kelvin (taken as ≈300 K, for the imaging temperature of 25–27 °C) and the microridge *L*_*p*_ was estimated from the experimental data. *Ε* is actually the Young’s Modulus that relates stress and strain within and *Ι*_*a*_ is the second moment of the cross-sectional area.

### Pattern wavelength

We estimated the overall microridge pattern wavelength for the yolk and flank cells in the Fourier domain. For this, we computed the 2D Fourier transform of the network-segmented logical images to determine the overall wavelengths of the microridge pattern within each cell. The 2D Fourier transform of the image *I(x,y)* was calculated using the Matlab *fft2* function, to compute,

22 $${I}_{f}\,\left({k}_{x},{k}_{y}\right)=\frac{1}{2\pi }\int {dx}\int {dy}\,{e}^{-i({k}_{x}x+{k}_{y}y)}\,I\left(x,y\right).$$

The Fourier space of *k*_*x*_ and *k*_*y*_ ranges from *-K* to *K*, where *K* = π/*∆s*, and *∆s* is the edge length of 1 pixel within the image. We defined the characteristic pattern wave number (*wn*) by,

23 $${wn}=\sqrt{\int d{k}_{x}\int d{k}_{y}\,{\left|\vec{k}\right|}^{2}\left|{I}_{{fN}}\,({k}_{x},{k}_{y})\right|},$$

where,

24 $$|\overrightarrow{k}|=\sqrt{{k}_{x}^{2}+{k}_{y}^{2}}.$$

The magnitude of the image Fourier transform was normalized given by,

25 $${I}_{{fN}}\,\left({k}_{x},{k}_{y}\right)=\frac{\left|{I}_{f}\,\left({k}_{x},{k}_{y}\right)\right|}{\sqrt{(\int d{k}_{x}\int d{k}_{y}\,\left|{I}_{f}\,({k}_{x},{k}_{y})\right|)}}.$$

The pattern wavelength λ, followed as

26 $$\lambda =\frac{2\pi }{{wn}}.$$

### Branch lengths

The network-segmented images were converted to logical arrays, and subsequently, a “skeleton operation” was used to obtain the traces of the microridges. From the image skeleton, we estimated branch lengths. Branch points were determined and subtracted from the trace images to obtain the skeletonized branches. Branch lengths were computed for each microridge branch by summing up their total number of pixels within a cell. We computed the mean branch lengths considering all the microridge branches for each segmented cell pattern.

### Velocity flow analysis

All the cells for this analysis were successfully tracked using a nearest neighbor distance cut-off ≤2.5 μm between cell centroids across time *t*_*n*_ and *t*_*n+1*_, which removed any image drifts and center of mass jitters. The optic flow analysis was carried out within the local rest frame of each cell. Cells underwent small-scale deformations due to local interactions with their neighboring cells. We estimated the microridge growth velocity (*v*_growth_) and the shrinkage velocity (*v*_shrinkage_). The growth velocity (*v*_growth_) inferred from the image encompassed the merging velocity of adjacent microridges and the local material assembly rates (leading to single microridge elongation events), while the shrinkage velocity (*v*_shrinkage_) accounted for the splitting velocity of microridges and the local disassembly rates (leading to single microridge fragmentation events); all four events are due to net actin dynamics within the microridges. We implemented an optic flow (Lucas–Kanade derivative of Gaussian) method using an inbuilt Matlab function to determine the velocities ($\vec{v}$) from a time series of spacing *Δt* from network-segmented cell patterns with intensity values *I* by computing,

27 $$\frac{\Delta I}{\Delta t}=\,\frac{I\left(t+\Delta t\right)-I\left(t\right)}{\Delta t}=\,-\vec{v}\cdot \vec{\nabla }I.$$

The image velocity divergence (${\mathcal{D}}$) was then computed as

28 $${\mathcal{D}}=\frac{\partial {v}_{x}}{\partial x}+\frac{\partial {v}_{y}}{\partial y}.$$

where ∂x and ∂y denote the spatial change in the x and y directions. We implemented a convolution operation using a smoothing kernel σ (where σ.*${\mathbb{1}}$, where σ = 1/9 and ${\mathbb{1}}$ is a 3 × 3 ones matrix) on the velocity component image matrices for subsequent computations to eliminate low-level noise within the image time series. We determined the regions of high positive divergence and strong negative divergence in the images and determined the velocities specifically at these locations. We then averaged the velocity over all such locations to determine the shrinkage velocities (*v*_shrinkage_) and growth velocities (*growth*), respectively. The mean growth velocity (*υ*_growth_) and mean shrinkage velocity (*v*_shrinkage_) were computed by averaging over the entire cell pattern and across all frames.

We computed the strain rate tensor (*S*) considering the symmetric component of the velocity gradient, given by,

29 $$S=\frac{1}{2}\left(V+{V}^{T}\right),$$

where the velocity gradient (*V*) is,

30 $$V=\left[\begin{array}{cc}\frac{\partial {v}_{x}}{\partial x} & \frac{\partial {v}_{x}}{\partial y}\\ \frac{\partial {v}_{y}}{\partial x} & \frac{\partial {v}_{y}}{\partial y}\end{array}\right].$$

The off-diagonal element of *S* are the shear strain rates (*γ*^*S*^) given by,

31 $${\gamma }^{S}=\frac{1}{2}\left(\frac{\partial {v}_{x}}{\partial y}+\frac{\partial {v}_{y}}{\partial x}\right).$$

The eigenvalues ${\varLambda}$_*1*_ and ${\varLambda}$_*2*_ of *S*, such that ${\varLambda}$_*1*_ > ${\varLambda}$_*2*_ were computed; ${\varLambda}$_*1*_ represent tensile strains, and ${\varLambda}$_*2*_ represent compressive strains.

We evaluated the overall principal strains by quantifying the magnitude of cell pattern strains, given by √2||S||, where ||S|| is the matrix norm within microridge regions only, ${\left\langle {\left\langle \sqrt{2}{||S||}\right\rangle }_{m}\right\rangle }_{t}$ such that ($\vec{\nabla }\cdot \vec{v}\,\ne\, 0$). We neglected the anti-symmetric part of the velocity gradient tensor, which represents rotational components, since localized rotational effects were not significant here on their own, as observed in the microridge flow patterns under wild-type conditions. The components of the S-tensor account for both shape and area-changing strain components.

### Area-deviatoric decomposition of the strain rate tensor

The decomposition of the 2D strain rate tensor (*S*) into area strain tensor (*S*^area^) and deviatoric strain tensor (*S*^dev^) can be written as,

32 $$S={S}^{{{\mathrm{area}}}}+{S}^{{{\mathrm{dev}}}}.$$

The *S*^area^ contains the area-changing, shape-preserving part, whereas the *S*^dev^ contains the shape-changing, area-preserving part of the total strain rate tensor (*S*).

The deviatoric tensor *S*^dev^ is a traceless second-order tensor (${tr}({S}^{{\mathrm{dev}}})=0$) given by,

33 $${S}^{\rm{dev}}=S-{S}^{\rm{area}},$$

where

34 $${S}^{\rm{area}}=\frac{1}{2}{tr}\left(S\right)\left[\begin{array}{cc}1 & 0\\ 0 & 1\end{array}\right].$$

The trace (*tr*) of the strain rate tensor (S) is the image velocity divergence $({\mathcal{D}})$ and also the *S*^area^ tensor is given by,

35 $${tr}\left({S}^{{{\mathrm{area}}}}\right)={tr}\left(S\right)={\mathcal{D}}=\vec{\nabla }\cdot \vec{v}.$$

The components of *S*^dev^ are purely deviatoric deformations. The eigenvalues of *S*^dev^ provide the deviatoric elongation (${\lambda }_{1}^{{{\mathrm{dev}}}}$) or deviatoric shrinkage (${\lambda }_{2}^{{{\mathrm{dev}}}}$) without change in the local area. The eigenvalues of ${S}^{{{\mathrm{area}}}}$ are both equal, and equal to *tr* (*S*^area^)/2.

### Measurement of fluctuations of intensity profiles within microridges

For this analysis, we cropped the center regions of cells within the yolk with relatively shorter microridges to obtain a temporal intensity profile within them. We used the detected 2D centroid positions after image segmentation. We wrote custom codes by implementing a multiple hypothesis tracking method frame-by-frame and solved using a linear-assignment problem (LAP) approach with a maximum distance cut-off of ~2.3 μm to find the same microridge in the next time frame. Briefly, a cost matrix was computed between centroids of detected microridges that considers all possible assignments between consecutive time frames and assignment is obtained by solving the matrix for minimal cost. A small distance cut-off ensured that tracking errors due to long microridges merging and splitting events were reduced considerably. The purpose of the tracking algorithm implementation was to compute the time evolution of the intensity profile along the microridges. For this computation, the pixel intensity and the eigenvector along the microridge length were used. Generic custom codes were written to obtain the dominant 2D eigenvectors (https://www.mathworks.com/matlabcentral/fileexchange/98894-image2dvectors) of each tracked microridge from the images. For any branched microridge, the intensity profile was assigned along the long branch, independent of microridge orientation within the image. We obtained the time evolution of the pixel coordinates [*x*_*p*_*, y*_*p*_] and the corresponding intensity readout (*I*_*pv*_) of microridges on the obtained microridge tracks, and then transformed coordinates into the frame spanned by their eigenvectors. The coordinate along the microridge *δ*_*l*_, is,

36 $${\delta }_{l}=\left({x}_{p}{y}_{p}\right)\cdot \left(\begin{array}{c}{e}_{1}\\ {e}_{2}\end{array}\right),$$

where *e*_*1*_ and *e*_*2*_ are the components of the normalized eigenvector that points along the length of the microridge. From the mean intensities, we computed an intensity profile along the 1D direction of the microridge, (*I*_*p*_), by summing the *I*_*pv*_ values for each discretized *δ*_*l*_.

### Local Gaussian curvature of the microridge pattern on periderm cell surfaces

We discretized the 2D space in (X, Y), and the Z height was taken as the microridge intensity (*I*) readout at each point on the periderm cell surface. We computed the first and second derivatives at each point and the first (*E, F, G*) and second (*L, N, M*) fundamental forms of the surface ${\mathbb{R}}$^3^. Then, the localized Gaussian curvature (*K*) was computed at each point on the cell surface that corresponded to the localized intensity within the microridges, given by,

37 $$K=\frac{{LN}-{M}^{2}}{{EG}-{F}^{2}}.$$

Gaussian curvature (https://www.mathworks.com/matlabcentral/fileexchange/11168-surface-curvature) was modified to compute the Gauss gradient with σ = 1.2 μm using (https://www.mathworks.com/matlabcentral/fileexchange/8060-gradient-using-first-order-derivative-of-gaussian) to extract the first and second derivatives at each point in the image.

### Quantitative parameter analyses of microridges from network-segmented images

Using the best-performing trained network, we segmented microridges of cell images with sizes of 256^2^ followed by rescaling to the physical size of image pixels for each image prior to parameter extraction. We considered 1502 cells from flank, yolk, and head regions comprising thousands of microridges for the estimation of persistence lengths (*L*_*p*_) of microridges. The comparative analysis of static parameters of microridges included the overall pattern wavelength (*λ*) and mean branch lengths (*<B*_*l*_>) on nearly equal numbers of cells from yolk and flank regions (300 and 293 cells) only. The movies for head regions were smaller in number and hence were not included in the comparative analysis and subsequent flow analyses to avoid any statistical bias. For the steady-state dynamic parameter analysis, we used the time series data from yolk and flank regions. We examined 9 movies with tracks of varying length durations between 11 minutes and 32 minutes. All the computational analysis was performed in Matlab (Mathworks) using custom codes.

### Reporting summary

Further information on research design is available in the Nature Research Reporting Summary linked to this article.

## Supplementary information

Reporting Summary

Supplementary Information

SupplementaryMovie1

SupplementaryMovie2

SupplementaryMovie3a

SupplementaryMovie3b

SupplementaryMovie4a

SupplementaryMovie4b

SupplementaryMovie5

SupplementaryMovie6

## Data Availability

The authors declare that the data supporting the major findings are available within the paper and its supplementary information files.

## Code availability

Computer codes have been provided as links within the Methods section. The codes for periderm cell segmentation, cell tracking, and microridges segmentation are available on GitHub (https://github.com/Bhavna-R/DeepLearningQuantAnalysActinMicroridges). Other additional codes can be provided upon reasonable requests to the corresponding author.
