# Supplementary material for: A deep learning framework for quantitative analysis of actin microridges
Source: NPJ Syst Biol Appl. 2023 Jun 2;9:21. doi: 10.1038/s41540-023-00276-7 (PMC10238495; doi:10.1038/s41540-023-00276-7)
Supplement: Supplementary file 1 — Reporting Summary [file 41540_2023_276_MOESM1_ESM.pdf]

nature portfolio

Corresponding author(s): DBPR NPJSBA-00820R1

Last updated by author(s): Mar 30, 2023

Reporting Summary

Nature Portfolio wishes to improve the reproducibility of the work that we publish. This form provides structure for consistency and transparency in reporting. For further information on Nature Portfolio policies, see our Editorial Policies and the Editorial Policy Checklist.

Statistics

For all statistical analyses, confirm that the following items are present in the figure legend, table legend, main text, or Methods section.

n/a	Confirmed	
☐	☒	The exact sample size (n) for each experimental group/condition, given as a discrete number and unit of measurement
☐	☒	A statement on whether measurements were taken from distinct samples or whether the same sample was measured repeatedly
☐	☒	The statistical test(s) used AND whether they are one- or two-sided<br>*Only common tests should be described solely by name; describe more complex techniques in the Methods section.*
☒	☐	A description of all covariates tested
☐	☒	A description of any assumptions or corrections, such as tests of normality and adjustment for multiple comparisons
☐	☒	A full description of the statistical parameters including central tendency (e.g. means) or other basic estimates (e.g. regression coefficient) AND variation (e.g. standard deviation) or associated estimates of uncertainty (e.g. confidence intervals)
☒	☐	For null hypothesis testing, the test statistic (e.g. F, t, r) with confidence intervals, effect sizes, degrees of freedom and P value noted<br>*Give P values as exact values whenever suitable.*
☒	☐	For Bayesian analysis, information on the choice of priors and Markov chain Monte Carlo settings
☐	☒	For hierarchical and complex designs, identification of the appropriate level for tests and full reporting of outcomes
☒	☐	Estimates of effect sizes (e.g. Cohen's d, Pearson's r), indicating how they were calculated

Our web collection on statistics for biologists contains articles on many of the points above.

Software and code

Policy information about availability of computer code

Data collection	Live images of the microridges on the periderm cells within the yolk, head and flank regions of the zebrafish embryo were recoded using a 40x dipping lens upright Zeiss confocal 880 microscope
Data analysis	All the computational analysis was performed in Matlab (Mathworks) using custom codes, in-built commands and already available open source codes (Methods, Supplementary Methods).

For manuscripts utilizing custom algorithms or software that are central to the research but not yet described in published literature, software must be made available to editors and reviewers. We strongly encourage code deposition in a community repository (e.g. GitHub). See the Nature Portfolio guidelines for submitting code & software for further information.

Data

Policy information about availability of data

All manuscripts must include a data availability statement. This statement should provide the following information, where applicable:

- Accession codes, unique identifiers, or web links for publicly available datasets
- A description of any restrictions on data availability
- For clinical datasets or third party data, please ensure that the statement adheres to our policy

The authors declare that the data supporting the major findings are available within the paper and its supplementary information files. Additional data that support the findings are given in Supplementary Tables.

Human research participants

Policy information about studies involving human research participants and Sex and Gender in Research.

Reporting on sex and gender	NA
Population characteristics	NA
Recruitment	NA
Ethics oversight	Department of Biological Sciences, Tata Institute of Fundamental Research, Colaba, Mumbai- 400005

Note that full information on the approval of the study protocol must also be provided in the manuscript.

Field-specific reporting

Please select the one below that is the best fit for your research. If you are not sure, read the appropriate sections before making your selection.

☒ Life sciences ☐ Behavioural & social sciences ☐ Ecological, evolutionary & environmental sciences

For a reference copy of the document with all sections, see nature.com/documents/nr-reporting-summary-flat.pdf

Life sciences study design

All studies must disclose on these points even when the disclosure is negative.

Sample size	We live imaged 8 embryos in 15 different regions (5, 6 and 4 movies on flank, yolk and head regions respectively).
Data exclusions	We manually checked for image segmentation quality by inspecting each pair of grayscale cell image with their annotated labels produced by the microridge segmentation algorithm and discarded any incorrectly segmented cells from the analyses.
Replication	Yes
Randomization	Random
Blinding	yes

Reporting for specific materials, systems and methods

We require information from authors about some types of materials, experimental systems and methods used in many studies. Here, indicate whether each material, system or method listed is relevant to your study. If you are not sure if a list item applies to your research, read the appropriate section before selecting a response.

Materials & experimental systems

n/a	Involved in the study	
☒	☐	Antibodies
☒	☐	Eukaryotic cell lines
☒	☐	Palaeontology and archaeology
☐	☒	Animals and other organisms
☒	☐	Clinical data
☒	☐	Dual use research of concern

Methods

n/a	Involved in the study	
☒	☐	ChIP-seq
☒	☐	Flow cytometry
☒	☐	MRI-based neuroimaging

Animals and other research organisms

Policy information about studies involving animals; ARRIVE guidelines recommended for reporting animal research, and Sex and Gender in Research

Laboratory animals	Zebrafish lineTg(actb1:GFP-utrCH)
Wild animals	NA

Reporting on sex	NA
Field-collected samples	zebrafish maintained by the fish facility at 28C
Ethics oversight	The zebrafish maintenance and experimental protocols used in this study were approved by the institutional (TIFR, Mumbai) animal ethics committee

Note that full information on the approval of the study protocol must also be provided in the manuscript.
