# Supplementary material for: A deep learning framework for quantitative analysis of actin microridges
Source: NPJ Syst Biol Appl. 2023 Jun 2;9:21. doi: 10.1038/s41540-023-00276-7 (PMC10238495; doi:10.1038/s41540-023-00276-7)
Supplement: Supplementary file 2 — Supplementary Information [file 41540_2023_276_MOESM2_ESM.pdf]

Supplementary Information

A deep learning framework for quantitative analysis of actin microridges

Rajasekaran Bhavna[1,2]*, Mahendra Sonawane[1]

1 Department of Biological Sciences, Tata Institute of Fundamental Research, Colaba, Mumbai- 400005

2 Current Address: Department of Data Science and Engineering, Indian Institute of Science Education and Research, Bhopal, Madhya Pradesh- 462066

*Corresponding author email: bhavnarajasekaran@yahoo.com

The supplementary information includes:

Supplementary Figures

Supplementary Figure Legends

Supplementary Table

Supplementary Table Legend

Additional Supplementary information includes 8 Supplementary Movies 1-6 (1-2, 3a-b, 4a-b, 5 and 6).

SUPPLEMENTARY FIGURES

Supplementary Fig 1.

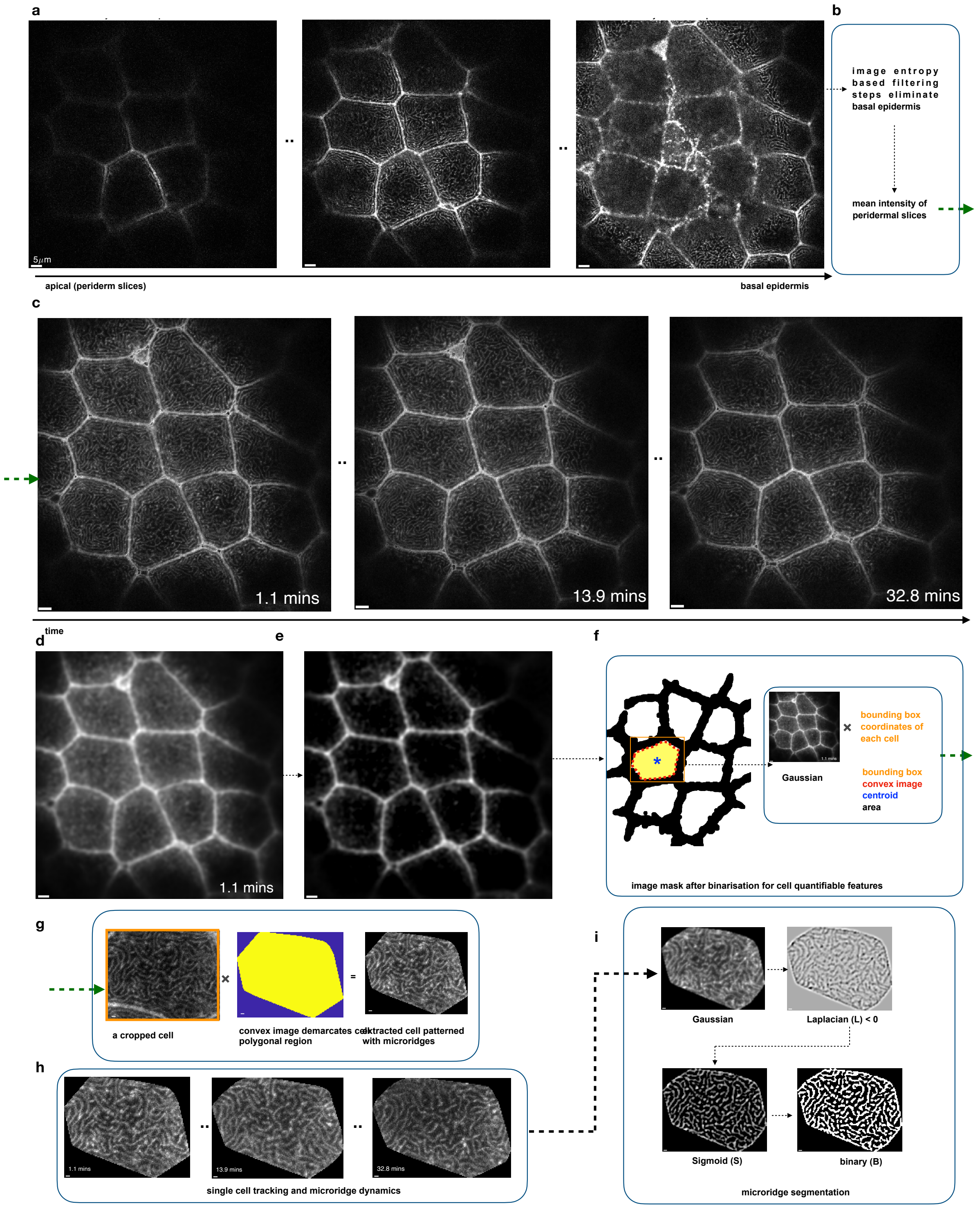

Supplementary Fig 1. **Image processing pipeline results in single cells patterned with microridges that forms the training set for the CNN approach**

a. Live imaging of zebrafish embryonic epidermis at 48hpf was performed to acquire images of periderm cells until 4-5μm depth reaching the basal epidermis. **b.** Gaussian image slices (σ=0.5 pixels) were processed using global entropy filtering to eliminate basal epidermis slices. For tackling periderm and basal signal on the same slice, a local entropy filtering threshold was used to eliminate the noisy signal from the basal slices. **c.** Time-lapse images of mean intensity periderm slice images. **d.** Gaussian convolved (σ=2.5 pixels) filtered periderm cells **e.** Processed images using a high pass frequency filter that eliminated only microridges pixels while preserving the cell membranes. **f.** Image binarization demarcated cell membrane boundary (red dotted line) and the following cell features were extracted: bounding box rectangular coordinates (orange); convex cell image (filled-in yellow); cell centroid (blue asterisk) and cell convex area. Product of bounding box rectangular coordinates of each segmented cell and the Gaussian image (in **b.**) extracted each cell within the box coordinates from the time-lapse images. **g.** Hadamard product of rectangular demarcated cell (with microridge) and each cell convex image extracted single cell images with their microridges. **h.** Centroid based cell tracking was implemented to follow the same cell over time and their microridge pattern dynamics. **i.** Microridge segmentation was achieved by employing sequential steps: Gaussian, followed by negative Laplacian image, processed further using a sigmoid function. Image binary was obtained from the sigmoid image. After a final manual segmentation quality inspection, 38% of cells were discarded to yield the labeled training set for the CNN approach. Details are given in Methods.

Supplementary Fig 2.

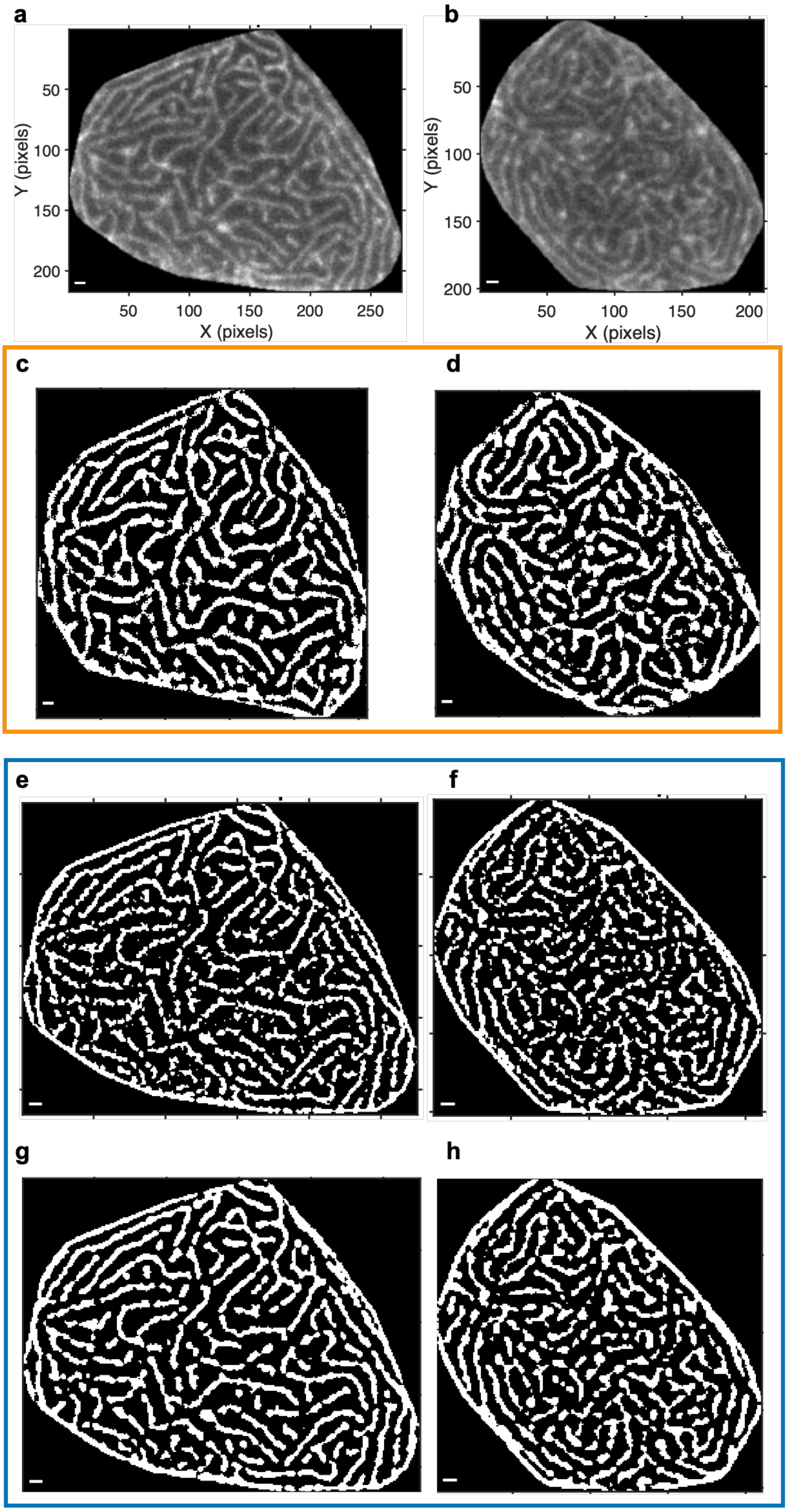

Supplementary Fig 2. Network versus conventional algorithm on unseen microscopy images produced at different SNR quality

a-b. Examples of two periderm cells from head region that were acquired with different microscopy settings hence different SNR quality (not part of the main dataset), scalebars indicate 1pixel to be 0.2131 µm and 0.1622 µm respectively after resizing images to 256×256. **c-d.** Pre-trained network performance on the unseen data shows reasonable segmentation quality. **e-f.** Grayscale images (a-b) processed with automated microridge labelling algorithm using default parameters (σ=0.7 pixels, ∂g=0.7 pixels, Methods, Eqs. 1, 4) led to over-segmentation effects and missing labels. **g-h.** Fine-tuning parameters to (σ=1 pixel, ∂g=0.9 pixels) in the microridge labelling algorithm was required to yield better segmentation results.

Supplementary Fig 3.

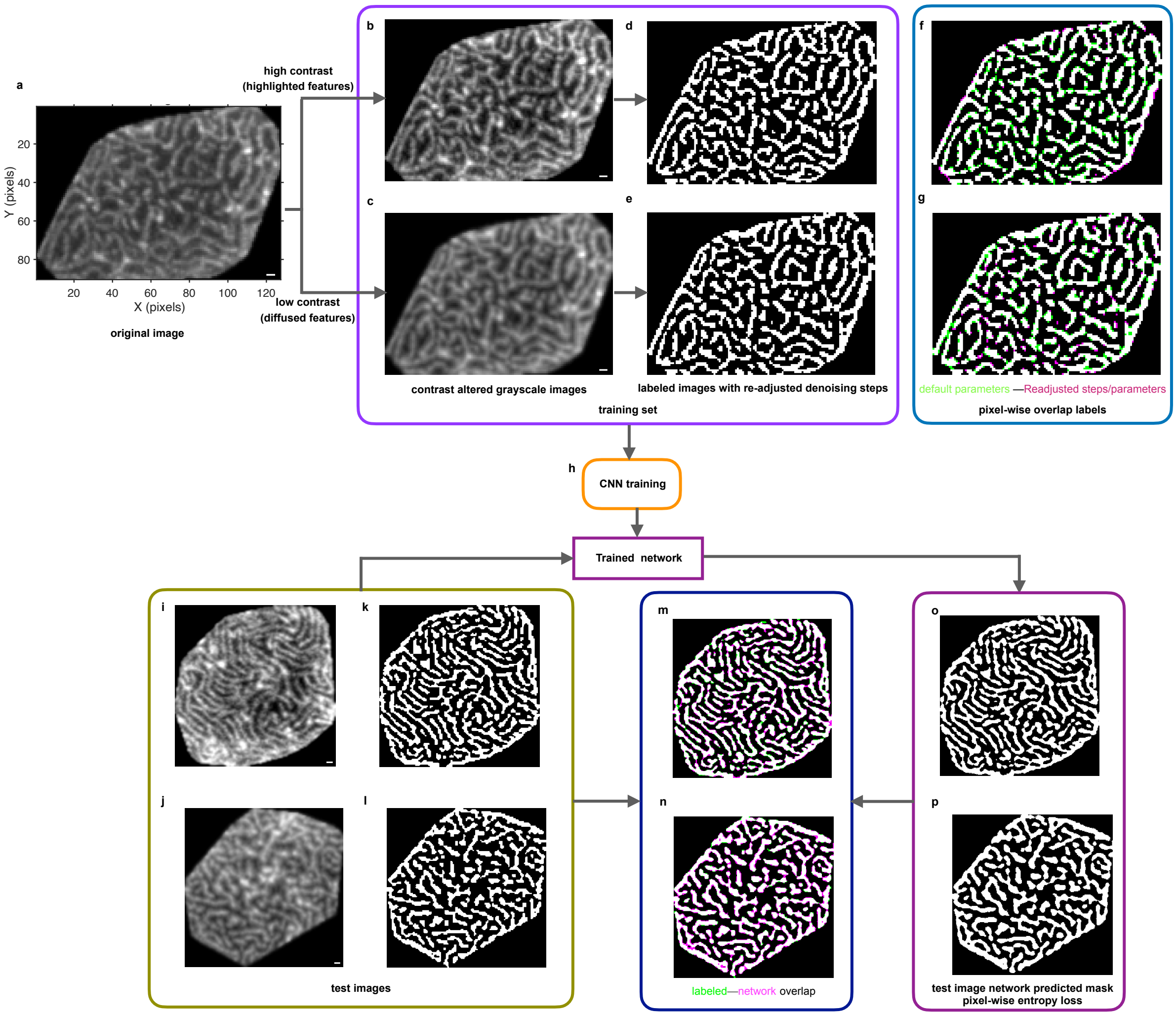

Supplementary Fig 3. CNN trained on artificially induced variations in local contrast within the images

a. Grayscale image of a yolk cell (scalebar indicates 1 pixel =0.1977 μm). **b.** Artificially induced high contrast grayscale image **c.** A relatively diffused low contrast images (compared to a.). **d.** Image in b. processed with re-adjusted parameters (σ=0.7 pixels, σ_g =0.5 pixels) in the microridge segmentation algorithm produced reasonable annotations. **e.** Image in c. processed with bilateral filtering followed by our microridge segmentation algorithm (σ=0.4, σ_g =0.5) produces correct pixel annotations **f.-g.** Pixel-wise labels using default parameters (σ=0.7, σ_g =0.7) that produced coarse/under-segmentation (green) on b.-c. respectively and using re-adjusted steps/parameters (pink, also shown in d-e). White labels indicate the overlap between both sets. **h.** CNN trained on both types of artificially produced high and low contrast images with their respective labeled annotations. **i.-l.** Two examples from the test dataset with high contrast and low contrast images (1 pixel is 0.0896 μm and 0.0942 μm after re-sizing images to 256×256 respectively) and their labelled annotations respectively. **m.-n.** Labelled outputs versus network predicted outputs for measuring network accuracy for high and low contrast images respectively. **o.-p.** Trained network predictions on test data in i-j respectively.

Supplementary Fig 4.

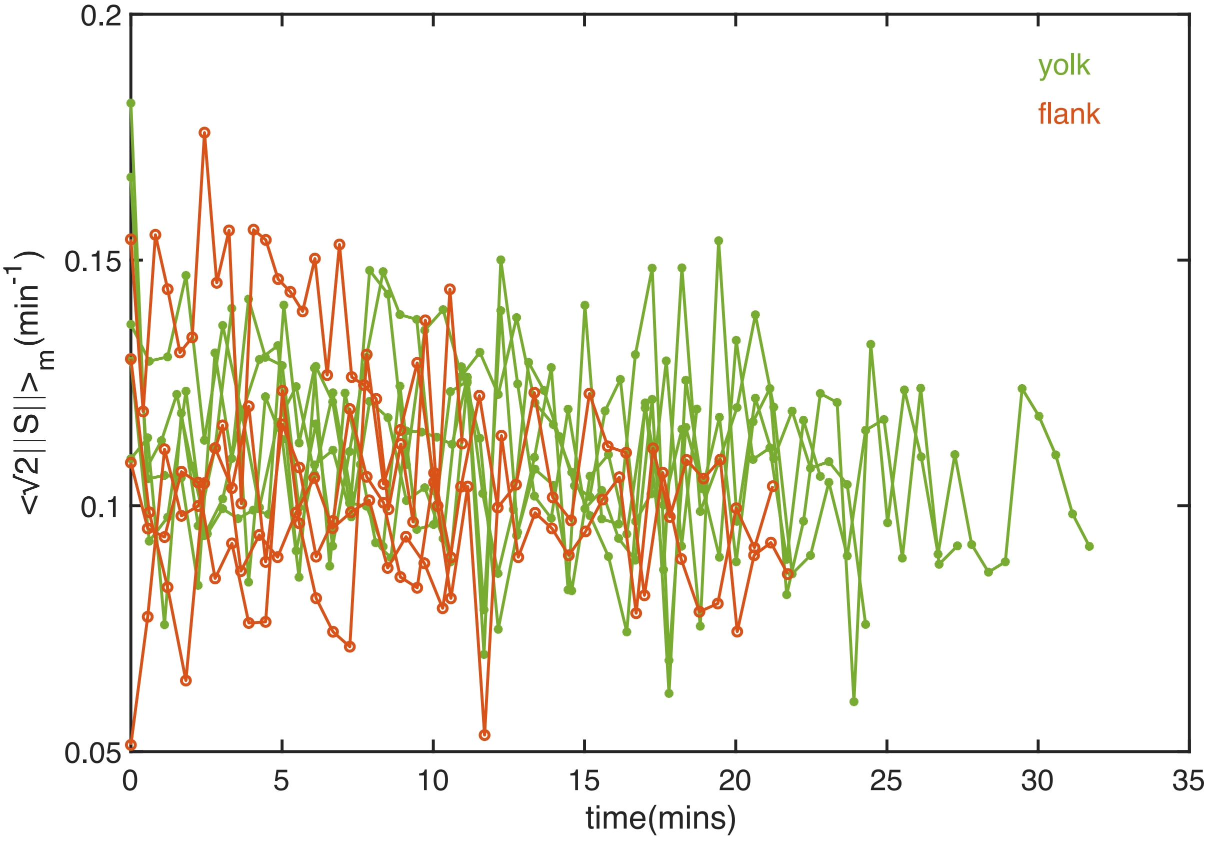

Supplementary Fig 4. Strain rate tensor norm of microridge cells patterns from yolk versus flank regions

Temporal evolution of the norm of the strain rate tensor averaged over microridge patterns, given by ($\sqrt{2}||S_m||$), (where *m* stands for microridge regions only) computed for yolk (green) and flank (orange) cell patterns. Generally, strain-related parameters of the yolk cell patterns are larger than in patterns from the flank regions. In both cases, the mechanical parameters of cell surface patterns revealed the presence of temporal periodic fluctuations indicating mechanical oscillations.

Supplementary Fig 5.

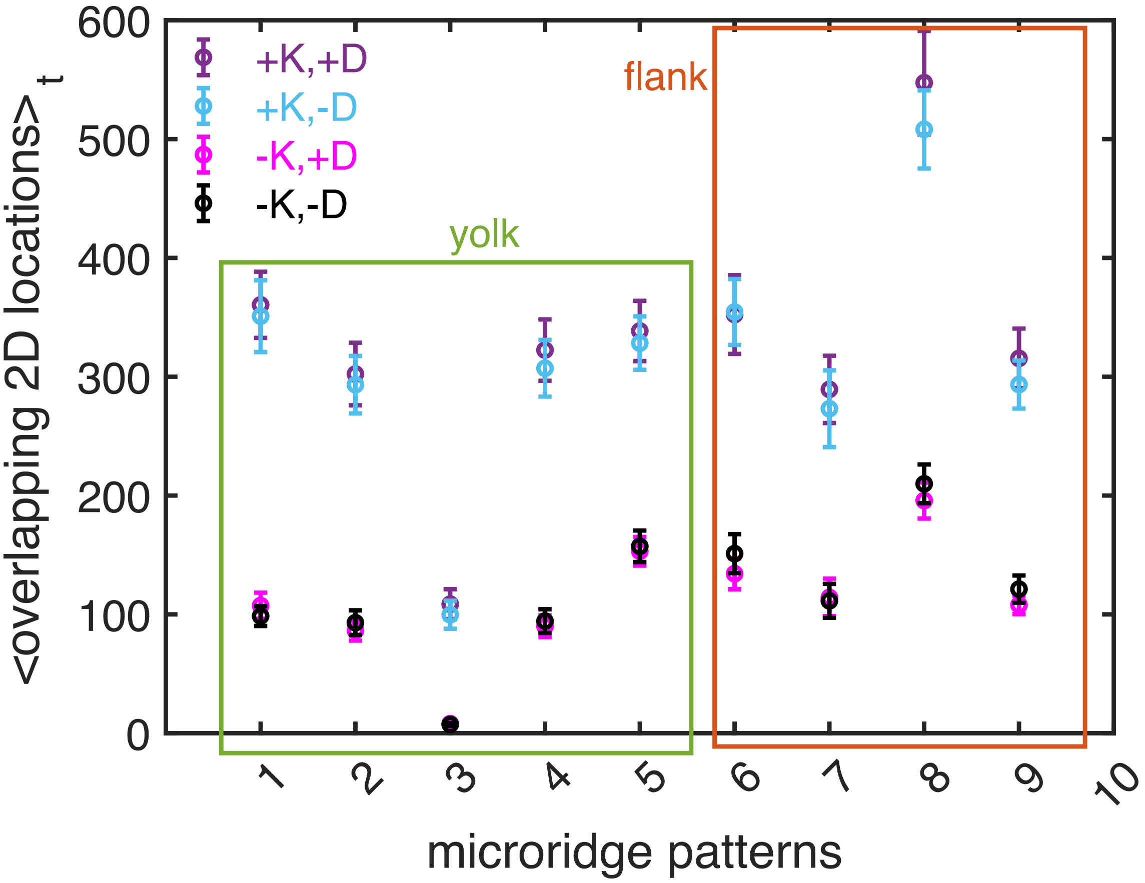

Supplementary Fig 5. 2D spatial co-occurrences of localized Gaussian curvatures (*K*) and the velocity field Divergence ($\mathcal{D}$) of the cell pattern dynamics

Each data point indicates the mean number of coincident 2D- locations and their standard error of mean for a cell pattern dynamics from yolk or flank region. In all cases, higher frequency counts of $+K$ locations at time t overlapped with $\pm\mathcal{D}$ locations from time t to $(t+1)$.

SUPPLEMENTARY TABLE

Supplementary Table 1.

No.	pattern	Duration (t) mins	v_{growth} ×10^{-2} μm/min	$v_{shrinkage}$ ×10^{-2} μm/min	$\langle\langle\sqrt{2}\|\|S_m\|\|\rangle\rangle_t$ ×10^{-2} min^{-1}
1	yolk	27.9	2.08±1.27	2.08±1.26	11.49±1.91
2	yolk	24.9	2.05±1.29	2.05±1.29	10.63±1.64
3	yolk (main)	23.3	2.33±1.39	2.34±1.39	11.15±2.16
4	yolk	32.2	2.10±1.30	2.10±1.31	10.64±1.92
5	yolk	19.2	2.13±1.36	2.14±1.37	11.08±1.78
6	flank	22.2	1.84±1.21	1.86±1.23	9.58±1.62
7	flank (supplementary)	11.1	2.04±1.34	2.05±1.35	9.77±1.64
8	flank	11.3	2.26±1.63	2.29±1.65	13.33±2.06
9	flank	21.8	1.89±1.18	1.90±1.19	9.79±1.41

Supplementary Table 1. Velocity flow analysis parameters for yolk and flank cell microridge patterns.

The parameters v_{growth}, $v_{shrinkage}$ and $\langle\sqrt{2}||S_m||\rangle$ were computed for 9 cell patterns. The overall strain rates built-up within the patterns of yolk are relatively higher than flank cell pattern as indicated by the computed parameters. The standard deviations are indicated for the temporally averaged quantities.
